# Synergistic Regulation of Plankton Communities by Polyculture of Grass Carp and Crucian Carp: Seasonal Dynamics and Interaction Networks Based on eDNA

**DOI:** 10.3390/biology15171468

**Published:** 2026-08-30

**Authors:** Liming Shao, Haipeng Guo, Yi Zhou, Haiqi Li, Wuhui Li, Chongqing Wang, Liang Guo, Kaikun Luo, Zhongyuan Shen

**Affiliations:** 1 Engineering Research Center of Polyploid Fish Reproduction and Breeding of the State Education Ministry, College of Life Sciences, Hunan Normal University, Changsha 410081, China; 2 Yuelushan Laboratory, Changsha 410128, China

**Keywords:** eDNA metabarcoding, polyculture system, plankton diversity, temporal variation, large-water ecological aquaculture

## Abstract

Large-water fisheries are central to China’s freshwater aquaculture, but their sustainability is threatened by eutrophication from traditional high-density stocking. Polyculture of grass carp and crucian carp offers an ecologically sound alternative; nevertheless, its direct effects on plankton communities require further study. We conducted monthly sampling from July to December 2023 (over two seasons within a single aquaculture cycle) in polyculture ponds and used eDNA metabarcoding (18S V9) with high-throughput sequencing. We detected 288 planktonic taxa across 28 phyla and 290 genera based on eDNA sequence assignment. Alpha diversity (Chao1, Shannon, Simpson) peaked in autumn and was lowest in winter, with Chao1 showing greater seasonal variation. Beta diversity revealed season as the dominant factor (R^2^ = 0.287, *p* = 0.012), with significant summer–winter divergence. Null-model analysis indicated stochastic processes (56.3%) slightly exceeded deterministic filtering (43.7%), and stochasticity was higher in summer (67.2%), suggesting fish bioturbation and grazing create heterogeneous microhabitats. Co-occurrence networks were dominated by positive links (87.3%), with *Cryptomonas* as a central hub, implying mutualism and niche complementarity. This study is the first to integrate eDNA metabarcoding with community assembly theory to elucidate seasonal succession and interactions in this polyculture. Our findings confirm that polyculture sustains diversity and stability via top-down (grazing) and bottom-up (nutrient release) effects, providing eDNA-based taxonomic evidence for management of large-water aquaculture.

## Simple Summary

In China, fish are often raised in large ponds at very high densities. This causes too many nutrients to build up in the water, leading to pollution and algal blooms. In this study, we tested a better method: raising grass carp and crucian carp together in the same pond (polyculture). We took water samples every month from July to December 2023 and used DNA technology to identify tiny plants and animals (plankton) in the ponds. We found that plankton diversity was highest in autumn and lowest in winter. Seasonal change was the main factor affecting which species were present. Interestingly, random events—like fish stirring up mud or eating selectively—had a slightly greater influence than environmental rules, helping create varied microhabitats. Most interactions among plankton were positive (they helped each other), with one type of algae acting as a key connector. The fish control plankton by grazing and recycle nutrients through their waste. This provides clear evidence that polyculture is a sustainable and environmentally friendly way to manage large-scale fish farms.

## 1. Introduction

Large water body ecosystems generally refer to inland waters such as natural lakes, reservoirs, and large artificial ponds with a surface area exceeding 0.5 km^2^. These systems are characterized by extensive open water, long water residence times, complex biotic community structures, and strong self-regulatory capacity [1]. In China, the fisheries output from large water bodies accounts for more than one-third of the total freshwater fishery production, serving as a crucial pillar for ensuring high-quality protein supply and increasing fishermen’s incomes. However, over the past decades, the extensive model of high-density feed-based aquaculture and indiscriminate pursuit of maximum yield has led to the discharge of large quantities of uneaten feed and metabolic waste into the water, triggering typical eutrophication symptoms such as frequent cyanobacterial blooms, sharp declines in dissolved oxygen, and expansion of hypoxic zones near the bottom. These problems have severely constrained the sustainable development of large-water fisheries [2,3]. In this context, transforming aquaculture practices and developing ecologically healthy farming models have become a consensus in the industry. Polyculture, which mimics the food-web structure of natural ecosystems, is widely considered an effective approach to alleviating aquaculture-derived pollution and enhancing ecosystem stability [4].

Plankton represent the most biologically active component of aquatic ecosystems, encompassing two major groups: phytoplankton and zooplankton. Phytoplankton, as primary producers, convert inorganic carbon, nitrogen, phosphorus, and other elements into organic matter through photosynthesis, thereby providing the energetic foundation for the entire food web. Zooplankton, as primary consumers, transfer matter and energy to higher trophic levels by grazing on phytoplankton, bacteria, and other micro-organisms [5,6]. Consequently, the community structure, population dynamics, and diversity of plankton directly influence the efficiency of material cycling, the pathways of energy flow, and the stability of the food web in the entire aquatic ecosystem [7,8]. Moreover, plankton are highly sensitive to changes in environmental variables such as temperature, nutrient concentrations, pH, and dissolved oxygen and are widely recognized as excellent bioindicators for assessing water quality [9]. From a fishery management perspective, plankton serve as both the initial prey for fish larvae and a supplementary food source for adult fish; their abundance and diversity directly determine the recruitment success and survival rates of fish populations. Therefore, plankton also provide essential reference data for establishing fishing quotas and designing stock enhancement and restocking strategies [10].

The impact of fish stocking on plankton communities is a classic research topic in aquatic ecology, with the underlying mechanisms generally categorized into two main pathways: the top-down effect and the bottom-up effect [11,12]. The top-down effect refers to the direct consumption of zooplankton or phytoplankton by fish through predation, which alters community structure and species composition. The trophic cascade theory proposed by Carpenter et al. [13] systematically elucidates this process, demonstrating that the presence of predators can exert cascading grazing pressure that propagates down through the food web, affecting lower trophic levels. The bottom-up effect, in contrast, operates through non-feeding pathways, such as the release of nitrogen, phosphorus, and other nutrients via fish excretion, the resuspension of sediment-bound nutrients through bioturbation, and the modification of underwater light conditions—all of which indirectly influence plankton growth rates and population proliferation [14,15]. These two effects often co-occur and interact, with their relative dominance depending on fish feeding habits, behavioral traits, stocking density, and ambient environmental conditions. Therefore, a scientifically sound assessment of the integrated effects of different fish stocking regimes on plankton communities holds both theoretical and practical significance for optimizing aquaculture structures, preventing algal blooms, and maintaining ecosystem health.

Grass carp (*Ctenopharyngodon idella*) and crucian carp (*Carassius auratus*) are the two most important freshwater aquaculture species in China. According to the 2025 China Fishery Statistical Yearbook, the production of grass carp in 2024 reached 6.165 million tonnes, accounting for 24.5% of the total freshwater fish output, while crucian carp production amounted to 2.819 million tonnes, representing 11.2%; together, these two species account for more than one-third of the total freshwater fish production [16]. Grass carp is a typical herbivorous fish, with adults feeding primarily on aquatic vascular plants, although juveniles (body length < 15 cm) also consume zooplankton and phytoplankton. Their feeding apparatus—comprising pharyngeal teeth and a horny pad—is adapted for shearing plant tissues, and their fine-structured gill rakers can intercept small planktonic organisms during filter-feeding [17]. Crucian carp is a classic omnivorous species with a broad diet that includes zooplankton (particularly cladocerans and copepods), benthic fauna, organic detritus, and some algae. Their gill rakers are relatively long and densely spaced, conferring high filtration efficiency, with a particular preference for larger zooplankton individuals [18]. In terms of spatial niche, grass carp predominantly occupy the upper and middle water layers, where they feed on aquatic macrophytes and macroalgae [17], whereas crucian carp are benthic dwellers that forage on zooplankton, benthic invertebrates, and organic detritus at the pond bottom [18]. This vertical segregation directly reduces dietary overlap because the primary food resources of the two species are distributed in different water layers: grass carp target plant materials available in the upper and middle water columns or along pond margins, while crucian carp exploit food resources associated with the sediment surface and benthic zone. Moreover, the benthic foraging activities of crucian carp—including sediment disturbance and re-suspension of organic matter—may indirectly enhance nutrient availability in the water column [14,15], which can stimulate phytoplankton production that serves as supplementary food for filter-feeding juvenile grass carp. However, this indirect trophic linkage does not constitute direct competition for the same food resources, as the two species operate at different trophic levels and spatial domains. Quantitatively, a previous study reported a niche overlap index of 0.6478 between grass carp and crucian carp in polyculture ponds [19], which, while indicating some degree of resource sharing, remains within a range that allows for stable coexistence. Consequently, the combination of distinct vertical habitat use and divergent feeding habits results in low dietary overlap, providing a solid ecological basis for their co-culture.

Chinese scholars initiated research on freshwater fish polyculture as early as the 1950s–1970s. During that period, polyculture systems based on the “four major domestic carps” (black carp, grass carp, silver carp, and bighead carp) were extensively promoted in the middle and lower reaches of the Yangtze River, yet these systems were originally designed primarily to maximize fish production, with relatively limited attention paid to ecological consequences [20]. Since the 1980s, researchers have begun to focus on food competition and complementarity among fish species within polyculture systems, as well as the effects of fish activities on the aquatic environment and plankton communities. For instance, Zhao [21] investigated the effects of common carp and a combination of silver carp and bighead carp on pond plankton, and found that filter-feeding by the latter could effectively control cyanobacterial biomass. Yang et al. [22] systematically compared the compositional changes in plankton and suspended particulate matter in polyculture ponds containing grass carp, silver carp, and common carp, revealing the differential impacts of various fish feeding strategies on suspended solids. Internationally, Milstein [23] provided a systematic review of interspecific interactions among fish in polyculture ponds, emphasizing the advantages of mixed stocking in enhancing system productivity. Turker et al. [24] compared the filtration rates of phytoplankton by tilapia and silver carp in aquaculture systems and found significant differences in filtration efficiency among species.

In the past two decades, ecological polyculture research has undergone substantial advancement. A systematic global review of fish polyculture literature spanning 55 years (1968–2023) revealed a notable shift from production-oriented studies toward sustainability-oriented approaches, including integrated multi-trophic aquaculture (IMTA), biofloc systems, and rice–fish ecosystems, with emerging research themes linking species diversity, nutrient recycling, and ecological efficiency [25]. Within China, researchers have increasingly employed quantitative ecological tools to assess polyculture performance. For example, Zhao et al. [19] used computer vision technology and niche analysis to compare grass carp–mud carp and grass carp–crucian carp polyculture modes, quantitatively evaluating niche overlap and competitive intensity between species. Nutrient budget studies in grass carp–crucian carp–bighead carp polyculture systems have demonstrated that zero-water exchange ponds can significantly reduce sediment accumulation rates of organic carbon and nitrogen while improving nutrient utilization efficiency [26]. Additionally, studies comparing grass carp monoculture and polyculture ponds using eDNA metabarcoding have revealed higher plankton diversity in polyculture systems [27,28].

In recent years, the “grass carp + crucian carp” polyculture model has been extensively demonstrated in major production areas such as Jiangsu, Hubei, and Hunan, yielding favorable economic returns [27,28] and overturning the traditional notion that these two species cannot be co-cultured. However, fundamental ecological studies, particularly systematic assessments targeting plankton communities, remain scarce. How exactly do grass carp and crucian carp jointly influence the plankton community? Which of the two effects—top-down or bottom-up—dominates? Do these effects vary consistently across seasons? These scientific questions have yet to be clearly answered.

It is noteworthy that traditional plankton surveys rely on morphological identification, a method that not only demands substantial taxonomic expertise from researchers but also suffers from long processing times and low throughput, with particularly severe shortcomings in detecting morphologically minute, rare, or cryptic species [29,30]. In recent years, the emergence of environmental DNA (eDNA) metabarcoding has revolutionized biodiversity research in aquatic systems [31]. This technique was first introduced by Rondon et al. [32] in 2000 for soil microbial studies; its principle involves extracting total DNA from environmental samples (water, sediment, soil, etc.), followed by PCR amplification of specific gene markers (e.g., 18S rDNA, *COI*) using universal primers and high-throughput sequencing, with species identification achieved through comparison against reference databases. Compared to morphological approaches, eDNA technology offers notable advantages, including high sensitivity (capable of detecting extremely low-abundance species), high throughput (hundreds of species can be identified in a single sequencing run), non-destructiveness (no need to collect organismal specimens), and a high degree of standardization [33,34]. A substantial body of comparative studies has demonstrated that eDNA-based methods generally outperform traditional net-collection and microscopic examination in species detection rates [35,36,37]. Nevertheless, eDNA technology also faces inherent limitations, such as primer bias (universal primers do not amplify all taxonomic groups evenly), incomplete reference databases (many microeukaryotes lack barcode sequences), and the fact that eDNA degradation rates are significantly influenced by temperature and pH [38,39]—factors that must be thoroughly considered when interpreting results. To mitigate these issues, we used the validated 18S V9 primer pair, multi-database BLAST (version 2.17.0) searches, and strict time/temperature controls during filtration and transport.

Against this background, the present study selected the grass carp–crucian carp polyculture ponds at the Qiaomaihu High-Quality Fish Research Institute in Hunan Province as the research site and employed eDNA metabarcoding to systematically investigate the species composition, diversity characteristics, and seasonal dynamics of the plankton community. Specifically, this study aims to: (1) characterize the species composition and seasonal dynamics of plankton communities in the grass carp–crucian carp polyculture system; (2) quantify the relative contributions of stochastic and deterministic processes to community assembly across seasons; and (3) infer the relative roles of top-down (fish grazing) versus bottom-up (nutrient release via excretion and bioturbation) effects in shaping plankton community structure. We tested two hypotheses: (a) the balance between top-down and bottom-up effects shifts seasonally, with top-down effects being more pronounced in summer when fish metabolic rates and feeding activity are high, and bottom-up effects becoming relatively more important in winter when fish activity is reduced; and (b) the combined effects of these two mechanisms sustain higher plankton diversity in the polyculture system than in monoculture systems, as reported in previous studies. The study aims to provide high-resolution taxonomic evidence to support the elucidation of the ecological mechanisms underlying grass carp–crucian carp polyculture, and to offer a scientific basis for the optimized management of ecologically sustainable polyculture systems. The four experimental ponds were stocked with different regimes: ponds A6 and A9 were polycultured with adult grass carp (*Ctenopharyngodon idella*) and crucian carp (*Carassius auratus*) at biomasses of approximately 12,137–15,657 jin (≈6093.5–8828.5 kg) and 19,830–25,212 jin (≈9915 kg–14,206 kg) per pond, respectively; ponds A2 and A3 were stocked only with crucian carp fry (400,000 and 7,000,000 individuals, respectively) for nursery purposes. Importantly, all ponds were managed under a “stocked and left to feed naturally” regime—no artificial formulated feed was supplied throughout the experimental period; fish relied entirely on natural food resources within the ponds. Specifically, we tested two hypotheses: (1) the relative importance of top-down versus bottom-up effects varies seasonally, with top-down effects (fish grazing) being more pronounced in summer when fish metabolic rates and feeding activity are high, and bottom-up effects (nutrient release via excretion and bioturbation) becoming relatively more important in winter when fish activity is reduced; and (2) the combined effects of these two mechanisms sustain higher plankton diversity in the polyculture system compared to monoculture systems, as reported in previous studies [36,37,38,39].

## 2. Materials and Methods

### 2.1. Study Site Description and Experimental Design

The study was conducted at the experimental base of the Hunan Qiaomaihu High-Quality Fish Research Institute in Yueyang City, Hunan Province (112°92′ E, 28°94′ N). The region has a mid-subtropical monsoon climate, with an annual mean temperature of approximately 17.1 °C, an annual mean precipitation of about 1400 mm, and an average frost-free period of approximately 270 days. The experiment lasted six months, from July (summer) to December (winter) 2023. Four identical standardized earthen ponds, designated A2, A3, A6, and A9, were selected as study units. Each pond was rectangular, with an area of approximately 120 mu (≈8 ha), an average water depth of 2.2 m, and a muddy bottom with earthen bank slopes. The ponds were hydrologically independent, each equipped with separate inlet and drainage systems. No water exchange was conducted during the experimental period, resulting in extremely low water exchange rates, which facilitated the observation of cumulative ecological effects of fish stocking. Moreover, the stocking regimes for the four experimental ponds were as follows: pond A2 was stocked with 400,000 crucian carp fry on 12 May 2023; pond A3 was stocked with 7,000,000 crucian carp fry on 5 May 2023; pond A6 was stocked with 19830 jin (≈9915 kg) of crucian carp and 12137 jin (≈6083.5 kg) of grass carp between 24 and 27 April 2023, with a grass carp to crucian carp biomass ratio of approximately 1:1.63; and pond A9 was stocked with 25,212 jin (≈12,606 kg) of crucian carp and 15,657 jin (≈7828.5 kg) of grass carp on 28 April 2023, with a biomass ratio of approximately 1:1.61. All ponds were managed under a “stocked and left to feed naturally” regime—no artificial formulated feed was supplied throughout the experimental period; fish relied entirely on natural food resources within the ponds. Individual body weights at stocking were not recorded; only total biomass per species per pond was documented. The crucian carp used in this study was the Hefang strain (*Carassius auratus*), a hybrid variety developed at Hunan Normal University. In Chinese aquaculture, (*jìyú*) broadly refers to fishes of the genus *Carassius*, but cultured populations—including the Hefang strain—are consistently identified as *C. auratus* in the scientific literature, distinct from the European *Carassius carassius*. We therefore retain *C. auratus* throughout this manuscript. Throughout the experiment, grass carp fed primarily on naturally growing aquatic macrophytes along the pond margins and on plankton within the ponds, while crucian carp foraged on benthic organisms, zooplankton, and organic detritus. Monthly routine monitoring was conducted to detect potential phytoplankton blooms through visual observation of water color, transparency, and surface scums—methods widely recognized as practical and effective for detecting abnormal algal blooms in aquaculture ponds [40,41]. To minimize observer bias and ensure consistency, all visual observations were performed by the same trained individual throughout the six-month sampling period. Throughout the study period, no visible surface scums, abnormal water discoloration, or extreme transparency values were observed, indicating the absence of abnormal algal blooms.

To provide environmental context for the study period, monthly air temperature data were obtained from the Yueyang Statistical Dataset (Yueyang Municipal Bureau of Statistics). During the study period (July–December 2023), mean monthly temperatures were as follows: July 29.9 °C, August 29.0 °C, September 25.3 °C, October 19.9 °C, November 14.0 °C, and December 7.2 °C, representing a complete seasonal transition from summer to winter. The annual precipitation in 2023 was 1176.2 mm, below the long-term average; July precipitation was 129.9 mm, 26.9% below the long-term average, and drought conditions persisted through the study period. For researchers interested in retrieving additional environmental variables, publicly available satellite remote sensing databases (e.g., NASA Earthdata, USGS Earth Explorer, Copernicus Open Access Hub) offer valuable resources for lake surface water temperature, water area, and chlorophyll-a concentrations in this region.

### 2.2. Water Sample Collection and Processing

Sampling was conducted once per month in mid-month, specifically on 15 July, 30 August (adjusted due to weather), 16 September, 18 October, 14 November, and 12 December, yielding a total of six sampling events. At each event, five water samples were collected from each pond using a five-point sampling method (four corners and the center) and pooled into a single composite sample, resulting in a total of 15 samples overall (sampling was not performed in certain months due to continuous heavy rainfall (August, ponds A2 and A3) or equipment malfunction—including pump failure causing water quality deterioration (A3, September–December) and sampling device breakdown (A6 in September and November; A9 in December); overall, 10 out of 24 pond-month samples were missed, but the remaining samples still provided adequate seasonal representation).

Prior to sampling, all contact materials were strictly sterilized. The 550 mL polypropylene sampling bottles were rinsed twice with ultrapure water, soaked in 10% bleaching powder solution for 10 min, and then rinsed three times with sterile ultrapure water before being air-dried for subsequent use. Forceps, scissors, and other tools were autoclaved (121 °C, 20 min) and dried. The 15 mL centrifuge tubes were sterile, individually packaged. Throughout the sampling process, personnel wore disposable sterile nitrile gloves. At each of the five sampling points per pond, a water sampler was used to collect approximately 550 mL of surface water (at a depth of 0.3 m); the sampling bottles were pre-rinsed twice with the sample water before filling. Immediately after collection, each water sample was placed in an insulated cooler with ice packs (maintained at approximately 4 °C). Upon completion of all samplings, samples were transported promptly back to the laboratory for processing, and the time from collection to filtration was controlled within 1 h to minimize biases arising from eDNA degradation and shifts in microbial activity.

### 2.3. Water Filtration and DNA Extraction

Upon return to the laboratory, each composite water sample (approximately 2750 mL, pooled from five 550 mL subsamples) was thoroughly homogenized and then divided into five aliquots of approximately 550 mL. Each aliquot was vacuum-filtered through a separate mixed cellulose ester membrane (0.45 μm pore size; Millipore, Burlington, MA, USA), resulting in five filters per pond per month. All filtrations proceeded without clogging, and the five filters from each composite sample were pooled for subsequent DNA extraction. Prior to use, the filtration apparatus was soaked in 10% sodium hypochlorite solution for 30 min and then rinsed thoroughly with sterile ultrapure water. For each filtration batch, an equivalent volume of sterile ultrapure water was filtered as a negative blank control to monitor potential exogenous DNA contamination during the experimental procedures. After filtration of each water sample, the membrane was carefully removed using sterile forceps, folded in half with the sample-bearing side inward, placed into a sterile 15 mL EP tube, and immediately stored at −20 °C. Total DNA enriched on the filter membranes was extracted using the Qiagen DNeasy PowerWater Kit (Qiagen, Hilden, Germany) following the manufacturer’s instructions. The main procedures included: (1) cutting the membrane into small pieces and placing them into a 5 mL centrifuge tube containing glass beads, adding 1 mL of PW1 lysis buffer preheated to 65 °C, and vortexing at maximum speed for 10 min to ensure complete cell lysis; (2) centrifuging at 3000× *g* for 1 min, transferring the supernatant to a new tube, sequentially adding 200 μL of PW2 solution and 650 μL of PW3 solution, incubating at 4 °C for 5 min, and centrifuging to remove impurities such as proteins and polysaccharides; (3) passing the entire supernatant through a spin column, followed by multiple washes with PW4 and PW5 wash buffers to remove residual contaminants; and (4) finally eluting the DNA with 60 μL of nuclease-free ddH_2_O, collecting the eluate in a sterile centrifuge tube, and storing at −20 °C for subsequent use. DNA concentration was determined using a Qubit 3.0 fluorometer (Thermo Fisher, Waltham, MA, USA) with the dsDNA HS assay kit, and DNA integrity was assessed by 1% agarose gel electrophoresis (120 V, 30 min), with band brightness and position visualized under a gel imaging system.

### 2.4. PCR Amplification and Product Purification

In this study, the eDNA extracted from each sample was amplified using universal primers targeting the V9 hypervariable region of the 18S rDNA gene (forward: TTGTACACACCGCCCGTCGC; reverse: CCTTCYGCAGGTTCACCTAC), a primer pair widely employed in plankton eDNA research and known to exhibit good amplification efficiency for both protists and metazoans [42]. The specific PCR reaction system and thermal cycling program are detailed in Table 1, and the amplification products were stored at 4 °C. Sterile ddH_2_O was included as a negative control. Each sample was amplified in triplicate, and the three replicate products were pooled and mixed thoroughly prior to electrophoresis detection. Subsequently, the amplified products were purified by gel excision using the AxyPrep DNA Gel Extraction Kit (AXYGEN, Union City, CA, USA), quantified by Qubit, and then sent to Shanghai Lingen Biotechnology Co., Ltd. (Shanghai, China). for paired-end sequencing on the Mova-Seq PE250 platform, with a target data volume of approximately 400,000 valid reads per sample.

### 2.5. Data Processing and Statistical Analysis

Raw sequencing data were first quality-filtered using Trimmomatic v0.39 to remove adapter sequences, reads with an ambiguous base (N) content exceeding 5%, and low-quality reads shorter than 100 bp. Paired-end reads were then assembled using FLASH v1.2.11 with a minimum overlap of 10 bp and a maximum mismatch rate of 0.1. The merged sequences were demultiplexed according to sample-specific barcodes (allowing up to one mismatch) and the read orientations were corrected accordingly. Denoising was performed using the DADA2 plugin within QIIME2 (version 2022.2) to remove chimeric sequences and generate amplicon sequence variants (ASVs). All downstream analyses were performed at the ASV level. The most abundant sequence within each ASV was selected as the representative sequence and subjected to BLAST alignment against the NCBI GenBank database and the Silva v138.1 database, with threshold criteria set at ≥97% similarity and ≥90% query coverage for taxonomic assignment. Taxonomic assignments were based on sequence similarity to reference databases and represent molecular detection of taxa rather than direct morphological identification of organisms. When conflicts arose between the two databases, species-level assignments prioritized NCBI GenBank (as Silva does not curate species-level taxonomy for 18S rRNA sequences); for conflicts at higher taxonomic levels, the conflicting assignments were manually verified by BLAST against the NCBI nt database, and the annotation with higher similarity, higher coverage, and better alignment statistics was retained. ASVs that failed to match any known species across all samples, as well as those detected in blank controls, were excluded to generate the final species abundance matrix.

Alpha diversity analysis was conducted using the vegan package (v2.6-4) in R (v4.2.1) to calculate the Shannon index, Simpson index, Chao1 index, and ACE index. Rarefaction curves were generated to evaluate the adequacy of sequencing depth. One-way analysis of variance (ANOVA) followed by Tukey’s HSD post-hoc test was used to compare diversity indices among seasons, with the significance level set at *p* = 0.05. Beta diversity was assessed based on the Bray–Curtis dissimilarity matrix and visualized via principal coordinate analysis (PCoA) and non-metric multidimensional scaling (NMDS). Differences in community structure among seasons were tested using the adonis2 function (PERMANOVA, with 999 permutations) in the vegan package. Species co-occurrence networks were constructed using the SparCC algorithm (sparcc package) on the top 70 genera by relative abundance, which cumulatively accounted for approximately 85–90% of the total relative abundance across all samples. This threshold was chosen to focus on the core community while excluding rare genera that may introduce spurious correlations due to low detection frequency and sequencing noise. Spearman’s correlation coefficients between genera were calculated. Edges were retained with a threshold of Spearman’s correlation coefficient >0.5 and *p* < 0.05, and network visualization was performed with Gephi v0.10.1. Topological properties (number of nodes, number of edges, average degree, clustering coefficient, and network diameter) were computed. Additionally, the null-model approach proposed by Stegen et al. [43] was applied to quantify the ecological processes governing community assembly; the relative contributions of deterministic and stochastic processes were estimated using the β-nearest taxon index (βNTI) and the Raup-Crick index (RC). Following Stegen et al. [43], we applied the following thresholds: |βNTI| > 2 indicates deterministic selection (βNTI > 2: heterogeneous selection; βNTI < −2: homogeneous selection); when |βNTI| < 2, RCbray > 0.95 indicates dispersal limitation, RCbray < −0.95 indicates homogenizing dispersal, and |RCbray| < 0.95 indicates undominated processes. All thresholds correspond to a 95% confidence level based on 999 randomizations.

## 3. Results

### 3.1. Plankton Community Composition and Seasonal Dynamics

**Figure 1:**
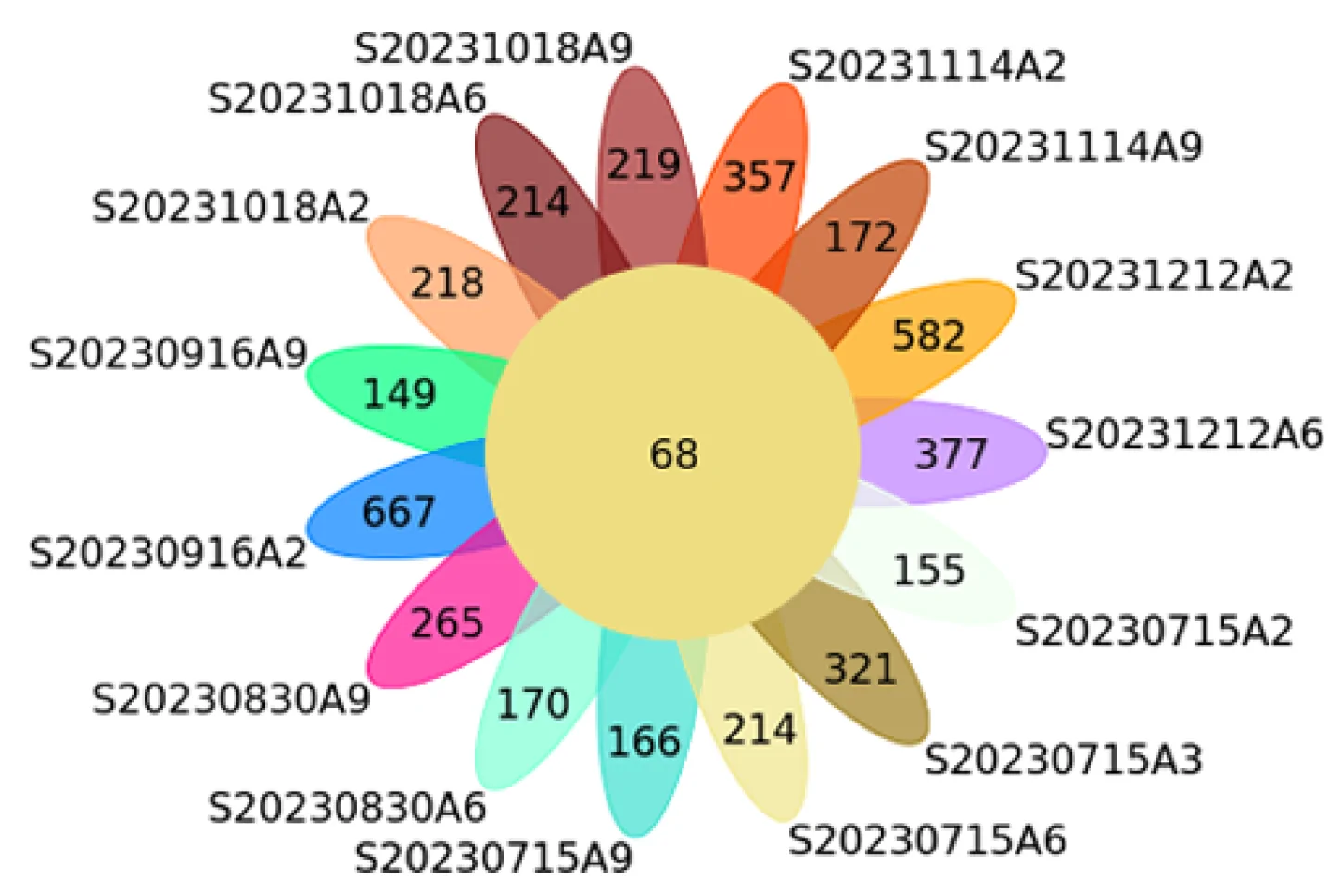
Distribution of plankton ASVs across samples.

High-throughput sequencing of the 15 samples yielded a total of 6,852,341 raw reads, which, after quality filtering and assembly, produced 5,635,185 valid reads (effective rate 82.2%), and a total of 8185 ASVs were obtained after clustering. The Venn diagram (Figure 1) revealed that only 68 ASVs (0.83% of the total ASVs) were shared across all samples, indicating extremely high compositional differences (i.e., high beta diversity) among samples. The number of unique ASVs varied markedly across samples, with sample S20230916A2 harboring the highest number of unique ASVs (667, accounting for 8.15%), suggesting that this sample possessed the most distinctive species composition. In terms of seasonal patterns, autumn samples exhibited the highest number of unique ASVs (1996), followed by summer (1291) and winter (959), which directly reflects the seasonal trajectory of a richer species pool in autumn and a substantial loss of species in winter.

**Figure 2:**
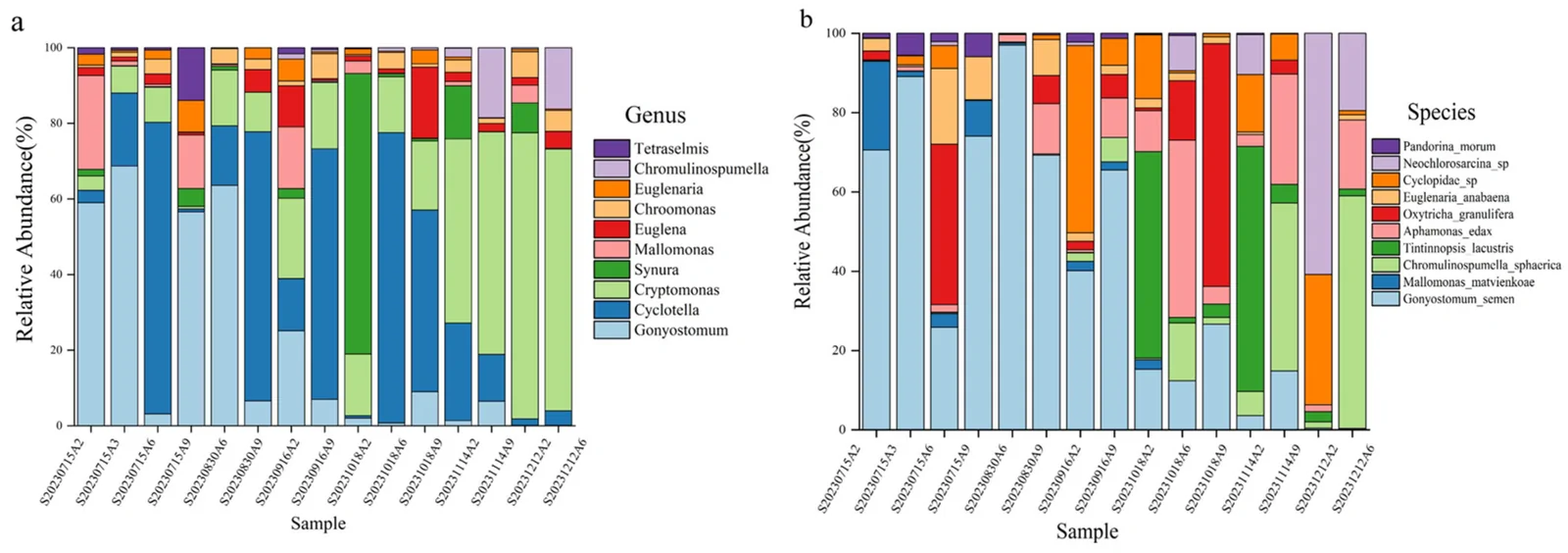
Taxonomic composition of plankton community based on eDNA sequencing. (**a**) Genus-level relative abundance; (**b**) Species-level relative abundance. The y-axis represents the relative sequence abundance (%) of each taxon; the x-axis denotes individual samples. Only dominant taxa are shown in the legend; low-abundance taxa are merged.

Through database comparison and taxonomic annotation, a total of 288 plankton species were identified, belonging to 28 phyla, 90 classes, 104 orders, 152 families, 290 genera (Table S1). At the phylum level, the top five phyla in terms of relative abundance were: Ochrophyta (mean relative abundance 32.5%), Chlorophyta (24.7%), Metazoa (15.3%), Cryptophyta (9.6%), and Cercozoa (6.8%), with all remaining phyla collectively accounting for 11.1%. At the genus level, a total of 24 genera exhibited mean relative abundances exceeding 1%, among which the top 10 most dominant genera were *Gonyostomum* (12.3%), *Cyclotella* (9.8%), *Cryptomonas* (8.5%), *Ochromonas* (6.2%), *Synura* (4.7%), *Trachelomonas* (3.9%), *Euglena* (3.2%), *Mallomonas* (2.8%), *Cyclops* (2.1%), and *Tintinnopsis* (1.6%) (Figure 2a). At the species level (Figure 2b), a total of 288 taxa were annotated, of which 19 species had relative abundances exceeding 1%. Over the entire year, *Gonyostomum semen* was the most dominant species (mean 9.2%), followed by *Cyclotella meneghiniana* (6.7%), *Cryptomonas curvata* (5.1%), and *Ochromonas variabilis* (4.3%).

From a seasonal perspective, the highest number of taxa was detected in autumn, with 227 taxa (78.8% of the total), followed by summer with 196 species (68.1%), and winter showing a sharp decline to 139 species (48.3%). This seasonal variation was not uniform across all taxonomic groups but rather exhibited clear group-specific patterns: the species richness of Ochrophyta and Chlorophyta peaked in autumn and decreased by approximately 40% in winter, whereas Metazoa (primarily rotifers and copepods) exhibited a more drastic reduction of 55% in winter. Taking pond A6 as a representative case to examine seasonal succession of dominant species: in summer (August), the most abundant species were *Oxytricha granulifera* (15.14%), *Chlorotetraedron incus* (12.81%), and *Gonyostomum semen* (9.69%); in autumn (October), the dominant species shifted to *Aphamonas edax* (12.31%), *Monomastix minuta* (10.91%), and *Nephroselmis olivacea* (9.55%); while in winter (December), *Chromulinospumella sphaerica* (30.93%) became overwhelmingly dominant, accompanied by *Neochlorosarcina* sp. (10.29%) and *Teleaulax amphioxeia* (10.16%). The species composition differed markedly among seasons, with an almost complete turnover of dominant species, reflecting a typical pattern of seasonal succession.

In addition, certain species exhibited strict seasonal specificity: *Ablabesmyia rhamphe*, *Absidia* sp., and *Synchaeta pectinata* were detected exclusively in summer; *Adineta vaga* increased in abundance in autumn but declined in winter; and *Ankyra judayi* showed a continuous decreasing trend over the course of the seasons. Such seasonal specificity of these species suggests that they may serve as potential indicator candidates for seasonal changes and fish predation pressure, although formal validation as ecological biomarkers would require concurrent environmental data and controlled experiments to disentangle the effects of temperature, nutrient availability, and grazing pressure. Future studies incorporating paired environmental measurements are needed to confirm their indicator value.

Within the same sampling batch, species composition also varied significantly among ponds. Taking August as an example, *Gonyostomum semen* reached a relative abundance of 90.17% in pond A6, forming an almost monospecific dominance; in contrast, this species accounted for only 26.87% in pond A9, where *Bicosoeca petiolata* (11.85%) and *Euglenaria clavata* (8.48%) occupied considerably higher proportions. Such inter-pond differences may be attributed to variations in the stocking regimes between the two ponds. Specifically, pond A6 was stocked with approximately 6083.5 kg of grass carp and 9915 kg of crucian carp (grass carp:crucian carp ≈ 1:1.63 by biomass), while pond A9 was stocked with approximately 7828.5 kg of grass carp and 12,206 kg of crucian carp (≈1:1.61 by biomass). The higher total stocking biomass in pond A9 (≈20.03 t) compared to pond A6 (≈16.00 t) may have contributed to the observed inter-pond variability in plankton community composition, reflecting the fine-scale regulatory effect of fish stocking structure on plankton communities.

### 3.2. Plankton Diversity Analysis

The calculated alpha diversity indices are summarized in Table 2. The Chao1 index ranged from 1093 (S20230715A9) to 2246 (S20230916A2), with a mean value of 1570; the ACE index ranged from 1093 to 2246, with a mean of 1571. The high consistency between these two indices indicates robust estimation of species richness. The Shannon index ranged from 4.74 (S20230715A9) to 8.47 (S20231018A9), with a mean of 7.19; the Simpson index ranged from 0.85 (S20230715A9) to 0.99 (S20231018A9), with a mean of 0.96. The highest values for both the Shannon and Simpson indices were observed in sample S20231018A9 (pond A9 in October), indicating that this sample not only harbored a relatively high number of species (195 species recorded) but also exhibited an exceptionally even distribution of abundance across species (Simpson index approaching 1, indicating low dominance).

When grouped by season, the mean Chao1 index was highest in autumn (1648.4 ± 327.3), followed by summer (1533.5 ± 241.3), and lowest in winter (1404.5 ± 57.3), with autumn values being significantly higher than winter (*p* < 0.05). Similarly, the Shannon index was highest in autumn (7.43 ± 0.85) and lowest in winter (7.17 ± 0.64); however, no significant difference was detected between summer and winter (*p* > 0.05). Notably, the seasonal variation amplitude differed between the two indices: the coefficient of variation (CV) for Chao1 across seasons (8.0%) was greater than that for Shannon (3.7%), suggesting that species richness is more sensitive to seasonal changes than community evenness.

**Figure 3:**
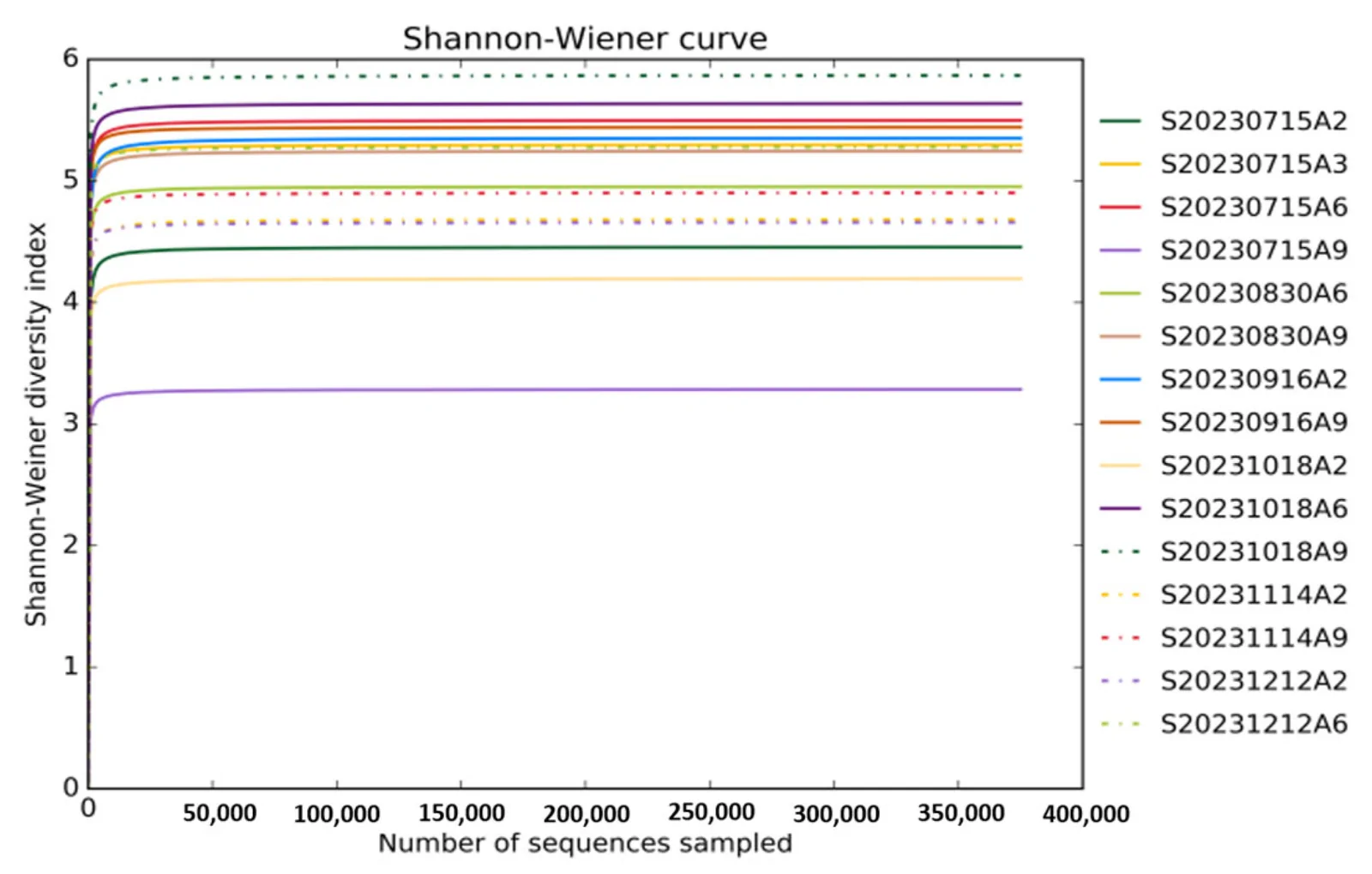
Rarefaction curves of plankton ASVs across all samples.

Rarefaction curves for all samples, generated using the ggplot2 package (Figure 3), all tended to approach saturation, and the coverage values ranged from 99.99% to 100%, indicating that sequencing depth was sufficient, with extremely few unobserved species, and that the present dataset could faithfully represent the plankton community composition of each sample.

**Figure 4:**
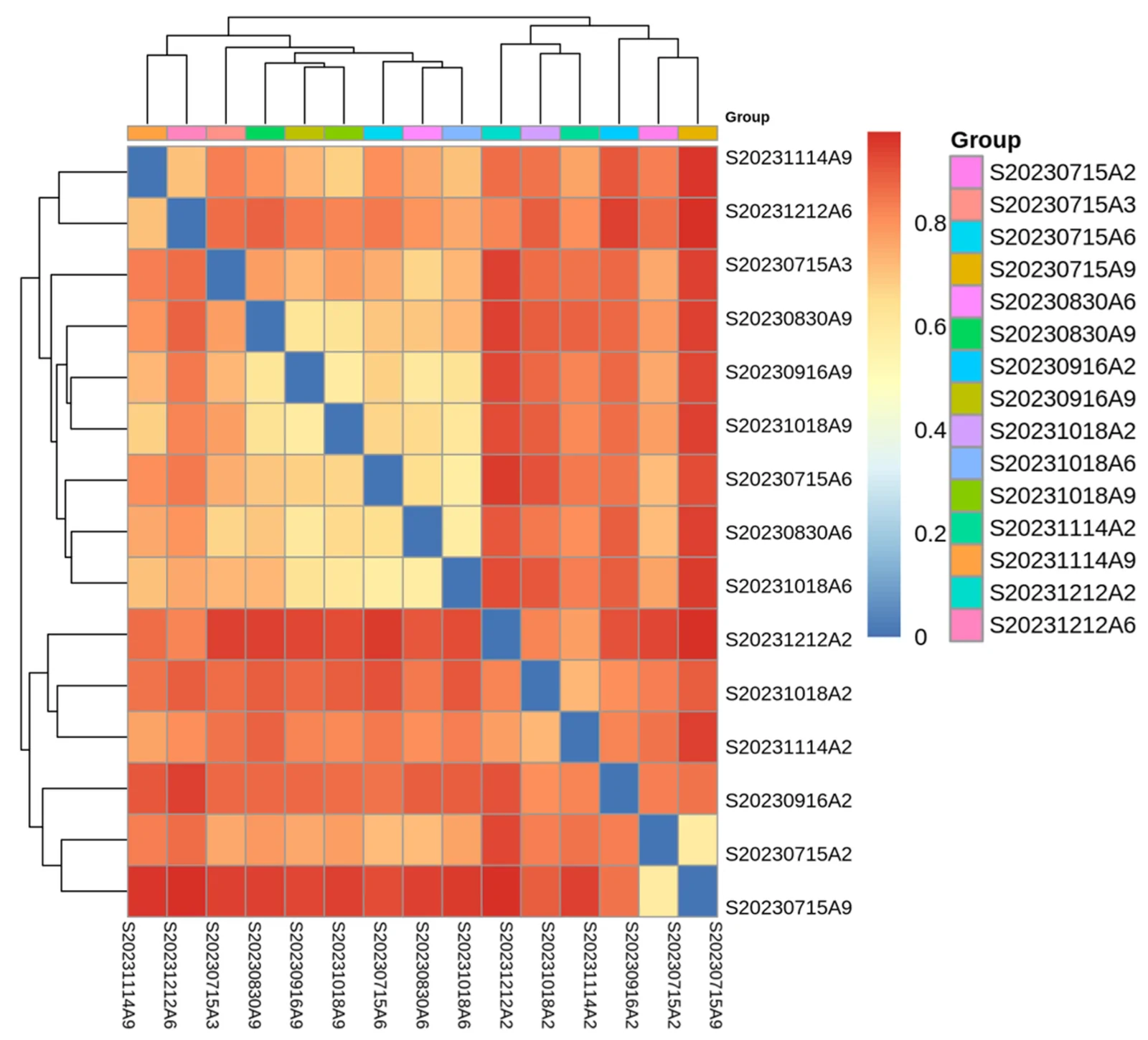
Pair-wise Bray–Curtis similarity heatmap of plankton community among all samples. The heatmap shows pairwise Bray–Curtis similarity values between each sample. Colour bar indicates the similarity coefficient (0–0.8). Hierarchical clustering was performed based on Bray–Curtis distance. The top coloured annotation bar represents sample grouping.

To visualize the dissimilarities among plankton communities, a distance matrix was constructed using the Bray–Curtis algorithm and plotted as a heatmap with the pheatmap package in R (Figure 4). The results showed that the dissimilarity indices between most sample pairs exceeded 0.5, and those between cross-seasonal samples often exceeded 0.8, indicating generally large compositional differences among samples. Cluster analysis revealed that most winter samples formed a distinct cluster, most autumn samples formed another cluster, while summer samples were more scattered, suggesting higher inter-pond heterogeneity during summer or a greater influence of stochastic processes.

**Figure 5:**
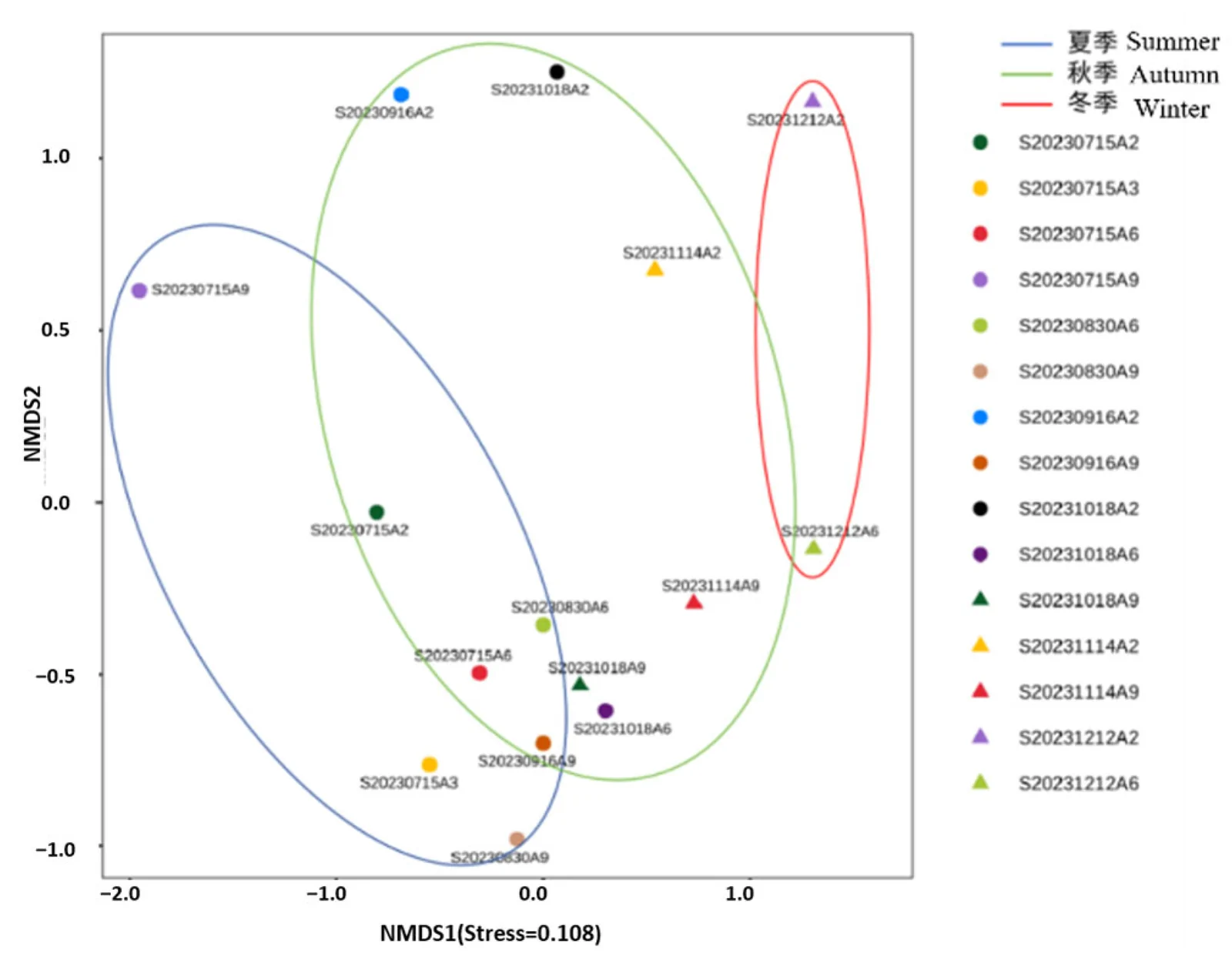
Non-metric multidimensional scaling (NMDS) ordination of plankton communities based on Bray–Curtis dissimilarities.

To further illustrate the spatiotemporal dynamics of the plankton communities, non-metric multidimensional scaling (NMDS) was performed based on the Bray–Curtis distance matrix (Figure 5). The NMDS analysis (stress value = 0.108, indicating good dimensionality reduction) showed that summer and winter samples were clearly separated along the first axis in the ordination space; autumn samples were positioned between summer and winter, partially overlapping with summer samples while separating from winter samples, suggesting a gradient transition pattern. PERMANOVA further quantified the seasonal differences: the season factor explained 28.7% of the community structure variation (R^2^ = 0.287, *p* = 0.012), indicating that season is one of the dominant factors driving community structure changes. Pairwise post-hoc comparisons revealed that summer and winter differed significantly (*p* < 0.05), whereas the differences between summer and autumn (*p* > 0.05) and between autumn and winter (*p* > 0.05) were not statistically significant, indicating that the major structural shift in the community occurred during the summer-winter transition.

### 3.3. Ecological Processes of Community Assembly

Based on phylogenetic null-model analysis, the βNTI values between sample pairs were calculated. The results showed that 56.3% of sample pairs had |βNTI| < 2, indicating that community assembly was primarily driven by stochastic processes (drift and dispersal limitation); whereas 43.7% of sample pairs had |βNTI| > 2, indicating that deterministic processes (environmental filtering and competitive exclusion) played a dominant role. Among the sample pairs dominated by deterministic processes, homogeneous selection (βNTI < −2) was predominant (34.1%), while heterogeneous selection (βNTI > 2) was less frequent (only 9.6%). These results suggest that in the grass carp–crucian carp polyculture system, although environmental factors exert a significant filtering effect, stochastic processes (e.g., historical contingency and dispersal limitation) also play a non-negligible role in community assembly. Further seasonal stratified analysis revealed that the contribution of stochastic processes in summer (67.2%) was significantly higher than that in autumn (48.5%) and winter (52.1%), likely attributable to the higher water temperatures, faster plankton growth rates, and the rapidly successional state of the communities during summer.

### 3.4. Species Co-Occurrence Network Analysis

**Figure 6:**
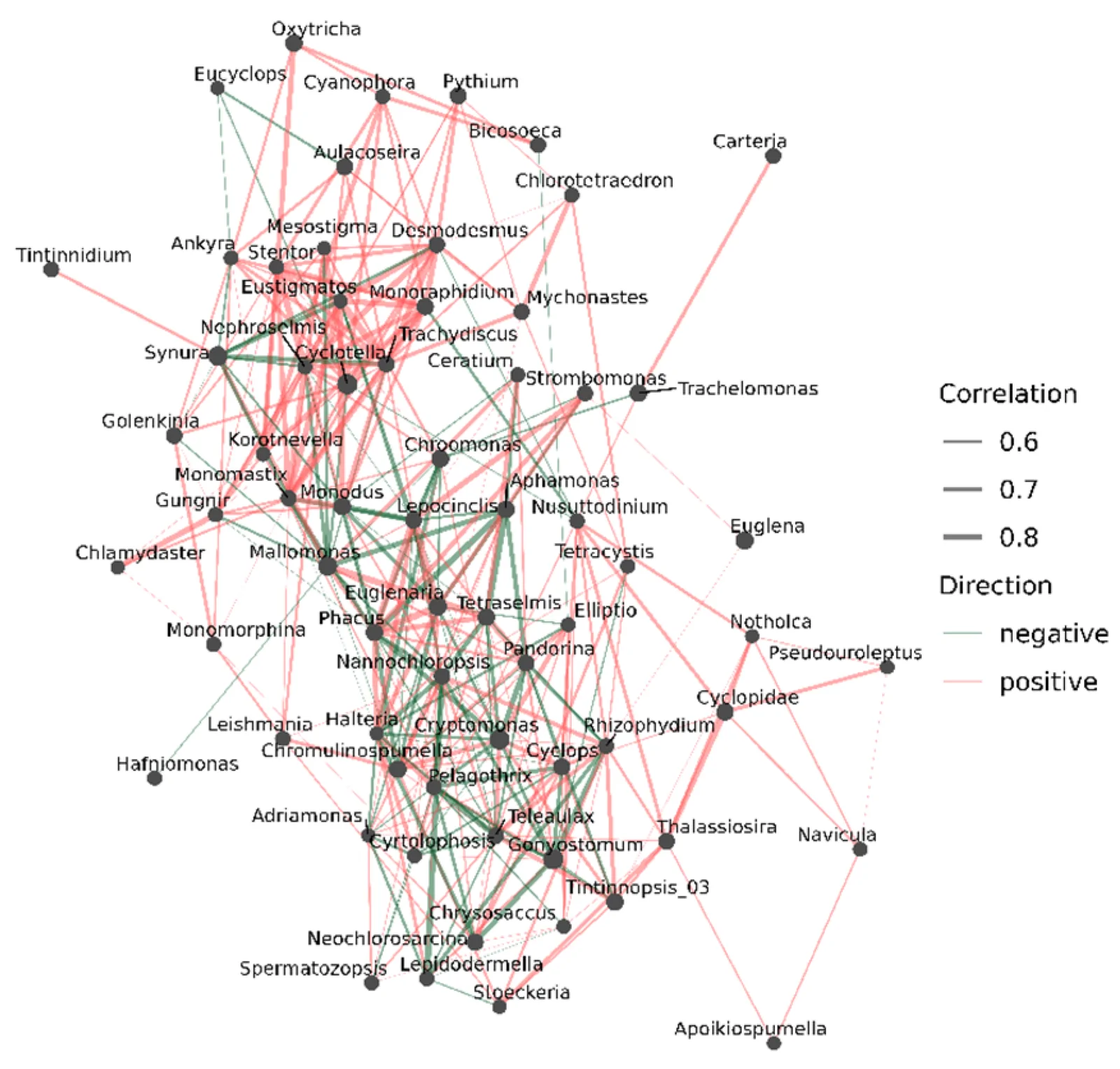
Species co-occurrence network of plankton communities at the genus level.

A co-occurrence network was constructed using the top 70 genera by relative abundance (Figure 6). The network comprised 67 valid nodes (three genera were filtered out due to no significant correlations with any other genera) and 332 significant edges (Spearman correlation coefficient > 0.5, *p* < 0.05), with a network density of 0.075, an average degree of 9.91, a network diameter of 4, and an average clustering coefficient of 0.213. Among the edges, 290 were positive (87.3%) and 42 were negative (12.7%), yielding a positive-to-negative edge ratio of approximately 6.9:1, indicating that synergistic interactions dominated within the community. The edge with the highest positive correlation was between *Trachydiscus* and *Eustigmatos* (Spearman’s ρ = 0.88), both of which are photosynthetic autotrophic ochrophytes, likely sharing similar environmental preferences or forming mutualistic microenvironments. The strongest negative correlation was between *Cyclotella* and *Cyclops* (Spearman’s ρ = −0.61), suggesting potential grazing pressure exerted by zooplankton on phytoplankton. Modularity analysis of the network (modularity = 0.45) identified five major modules, with the largest module consisting of 22 nodes, primarily algae from Ochrophyta and Chlorophyta, reflecting tight reciprocal associations among photosynthetic autotrophs. The genus with the highest degree centrality was *Cryptomonas* (degree = 27), indicating its central hub position in the community interaction network, likely linking to numerous other taxa through the provision of organic carbon sources or by fostering microbial microenvironments.

## 4. Discussion

The present study revealed a distinct seasonal pattern of plankton communities, characterized by an “autumn peak and winter trough,” a phenomenon resulting from the combined effects of temperature, light, nutrients, and biological factors (fish predation). Temperature, as the most fundamental environmental driver, not only directly affects plankton growth rates and reproductive efficiency (Q10 effect) [44], but also indirectly alters light availability and nutrient supply by regulating the intensity of thermal stratification and mixing depth. Although systematic environmental measurements were not conducted in this study, based on the climatic characteristics of the study region—a subtropical monsoon climate with substantial annual temperature fluctuations—it is reasonable to infer that seasonal changes in water temperature are the primary factor driving community succession. High summer temperatures (typically >28 °C), while theoretically conducive to rapid algal proliferation, may on the one hand inhibit the growth of certain cold-water species, thereby reducing species evenness; on the other hand, intense summer irradiance and thermal stratification can lead to nutrient depletion in surface waters, limiting the available niche space for competitively inferior species. As autumn arrives, both air and water temperatures decline to more favorable ranges (15–25 °C), vertical mixing intensifies, nutrient replenishment from bottom waters increases, and fish feeding intensity decreases due to reduced metabolic rates. The superposition of these multiple favorable factors renders autumn the season with the highest plankton species richness and diversity [45,46]. In winter, when water temperatures drop below 10 °C, plankton metabolic rates decrease markedly; some species form resting cysts and sink into the sediment, and shortened daylength and reduced light intensity further contribute to a substantial loss of species and a simplification of community structure. This seasonal pattern is highly consistent with the classic description of plankton seasonal succession in temperate lakes proposed by Sommer et al. [45] in the PEG (Plankton Ecology Group) model, indicating that natural seasonal rhythms remain the most fundamental drivers shaping plankton communities even in managed aquaculture ponds. Notably, the magnitude of seasonal responses varied considerably among different taxonomic groups. In this study, the decline in zooplankton species richness in winter (55%) was greater than that of phytoplankton (approximately 40%). This is likely because zooplankton, as heterotrophs, depend more directly on phytoplankton as a food resource, and the cascading effects along the food chain amplify the impact of reduced primary productivity in winter [47]. Furthermore, *Gonyostomum* attained extremely high abundances in some ponds during summer (up to 90% in pond A6) but disappeared completely in winter, indicating that this genus employs a distinctly “opportunistic” strategy—rapidly occupying dominant niches under favorable conditions of high temperature and abundant nutrients during summer, yet lacking tolerance to low temperatures, and thus retreating rapidly when environmental conditions deteriorate [48,49,50]. This near-complete turnover of dominant species between summer and winter reflects a fundamental ecological strategy: different taxa exhibit distinct thermal optima and life-history trade-offs. Summer dominance by *Gonyostomum semen* is consistent with its competitive advantages under warm, stratified conditions: its flagella enable vertical migration to access light and nutrients; its mixotrophic capacity provides a competitive edge when inorganic nutrients are limiting; and its large cell size reduces grazing losses [51,52,53]. Its near-disappearance in winter reflects temperature-sensitive physiology and the energetic cost of maintaining flagella and mixotrophic capacity under low temperatures and reduced light. Conversely, winter dominance by *Chromulinospumella sphaerica* likely reflects its ability to sustain growth at low temperatures, its tolerance of low light, and its reduced metabolic costs, allowing it to occupy the vacant niche left by summer specialists [54]. This seasonal species replacement, rather than coexistence, suggests that environmental filtering is the predominant force operating over seasonal timescales, with different ‘winning’ traits selected under different seasonal conditions.

The higher inter-pond heterogeneity observed during summer can be explained by the interplay between species’ dispersal capacities and localized environmental conditions. Summer’s warm temperatures and abundant resources promote rapid population growth, allowing stochastic colonization events to be amplified rather than homogenized by competitive exclusion, especially when different ponds experienced slightly different initial colonizer assemblies. Additionally, this heterogeneity may reflect the spatial patchiness of fish bioturbation and grazing pressure across ponds. Grass carp and crucian carp, with their different feeding modes and behaviors, create locally distinct nutrient regeneration patterns and selective predation pressures, leading to divergent community trajectories across ponds during summer. Our data support this: summer samples showed the greatest inter-pond variation in dominant species (e.g., *Gonyostomum semen* reached 90% in pond A6 but only 27% in pond A9), whereas autumn and winter samples were more compositionally similar across ponds, consistent with the homogenizing influence of lower temperatures and reduced fish activity.

The higher contribution of stochastic processes in summer (67.2%) compared to autumn (48.5%) and winter (52.1%) can be mechanistically explained by the combined effects of high temperature and intensified fish activity. First, elevated summer temperatures (monthly means 29.0–29.9 °C) accelerate plankton growth rates and generation times, allowing rapid population responses to localized disturbances; this creates a ‘lottery’ dynamic where early colonizers or locally favored genotypes disproportionately shape community composition before competitive exclusion can homogenize them. Second, the increased metabolic rates and feeding activity of both grass carp and crucian carp under warm conditions [17,18] intensify bioturbation (sediment resuspension) and selective grazing, generating transient, patchy microhabitats that promote stochastic dispersal and establishment of taxa rather than enforcing deterministic environmental filtering. Third, the vertical segregation of the two fish species—grass carp in upper layers, crucian carp in benthic zones—may differentially affect different plankton compartments, adding further stochasticity to community assembly. In contrast, during winter, reduced fish activity and lower temperatures slow ecological processes, allowing deterministic selection (e.g., survival of cold-tolerant taxa) to become relatively more important. This seasonal shift in the balance of stochastic and deterministic processes provides a mechanistic framework for understanding community dynamics in polyculture systems.

However, we acknowledge that direct measurements of water temperature, light intensity, and nutrient concentrations were not performed in this study. Consequently, our interpretations of seasonal succession as being driven by these factors are based on established ecological principles [45,46] and indirect evidence (e.g., regional air temperature data, known seasonal patterns of light availability, and inferred nutrient dynamics from fish excretion and bioturbation [14,15]), rather than site-specific environmental data. This limits our ability to quantitatively partition the relative contributions of individual environmental drivers. However, the primary objective of this study was not to quantify environmental drivers per se, but to assess plankton community dynamics in response to the polyculture regime. The seasonal patterns we observed—consistent across multiple ponds and independent of absolute environmental measurements—remain robust and support our core conclusions. Future studies combining high-frequency environmental monitoring with eDNA metabarcoding would be valuable to further disentangle the specific roles of temperature, light, and nutrients in driving community succession.

We also acknowledge that our sampling design was unbalanced, with pond A3 sampled only once after July due to pump malfunction that caused water quality deterioration. This limitation precluded the use of formal mixed-effects or repeated-measures models to separate pond-specific effects from seasonal effects. To minimize bias, pond A3 was excluded from all seasonal comparative analyses and was only included in the overall species inventory. Our primary analytical methods for seasonal comparisons—PERMANOVA and NMDS—are distance-based permutation methods that remain valid with unbalanced designs [55,56]. Furthermore, each season had at least two independently sampled ponds (summer: four ponds in July plus A6/A9 in August; autumn: A2/A9 in September, A2/A6/A9 in October, A2/A9 in November; winter: A2/A6 in December). The consistency of seasonal patterns across these independent ponds supports the robustness of our seasonal interpretations. Future studies with balanced repeated-measures designs would be valuable to further validate these findings.

Our interpretation of the observed seasonal patterns is grounded in the theoretical framework of top-down and bottom-up regulation, which we apply to the grass carp–crucian carp system as follows. The stocking of grass carp and crucian carp exerts influence on plankton communities through two pathways: the top-down effect (direct predation) and the bottom-up effect (nutrient input and sediment disturbance), with the relative strength of these two effects varying dynamically with season and pond conditions [11,12,13,14,57]. Regarding the top-down effect, both juvenile and adult grass carp can filter small planktonic organisms through their gill rakers; although grass carp primarily feed on aquatic macrophytes, the proportion of plankton in their diet increases significantly during seasons when plant food is scarce (e.g., winter) or during juvenile stages [17]. Crucian carp, as an omnivorous species, show a clear feeding preference for zooplankton, particularly cladocerans and copepods [18]. In this study, the relatively low abundance of large zooplankton such as *Cyclops* during summer and autumn may primarily reflect the grazing pressure exerted by crucian carp, although other factors such as summer diapause and interspecific competition with cladocerans may also contribute to this pattern. The direct consequence of the top-down effect is a reduction in the abundance of certain sensitive groups; however, such grazing pressure can also generate a “release effect”—that is, by reducing zooplankton grazing on phytoplankton, phytoplankton biomass may instead increase [12].

In terms of the bottom-up effect, the feces of grass carp, which feed on plants, are rich in incompletely digested plant detritus and dissolved nutrients (especially phosphorus), which are rapidly utilized by phytoplankton once released into the water column, as reported in previous studies [58,59]. Meanwhile, the activity of both grass carp and crucian carp on the pond bottom—including the turning over of sediment by grass carp while feeding on benthic plants and the disturbance caused by crucian carp during benthic filter-feeding—resuspends sediments and accelerates the release of nutrients such as nitrogen and phosphorus from the sediment into the overlying water [59]. This bioturbation effect is particularly pronounced in shallow aquaculture ponds; some studies have shown that benthic activities of crucian carp-like fish can increase nutrient fluxes across the sediment-water interface by two- to three-fold [59]. It is important to emphasize that in the grass carp–crucian carp polyculture system, the top-down and bottom-up effects do not operate independently but are coupled through complex feedback mechanisms: phytoplankton proliferate in response to the bottom-up effect, which is subsequently followed by zooplankton population growth (with a time lag); the increased zooplankton abundance in turn attracts greater grazing pressure from crucian carp, ultimately resulting in a dynamic equilibrium. Based on the observed seasonal succession patterns and the couling between top-down and bottom-up effects, we propose that such feedback mechanisms may contribute to maintaining community diversity and stability in the polyculture system over seasonal timescales. However, we acknowledge that this interpretation remains a testable inference rather than a directly demonstrated conclusion, as our study duration (six months) does not allow for assessment of inter-annual dynamics or long-term stability. Multi-year monitoring or controlled mesocosm experiments would be valuable to further validate these proposed mechanisms and to assess whether the observed feedbacks indeed confer stability over extended timescales.

This distinguishes the grass carp–crucian carp system from the traditional filter-feeding polyculture model dominated by silver carp and bighead carp, in which the bottom-up contribution is relatively smaller. Silver carp and bighead carp primarily suppress phytoplankton directly through efficient filter-feeding (a strong top-down effect) with minimal sediment disturbance [60]; in contrast, the frequent bioturbation by grass carp and crucian carp facilitates more active internal nutrient release, making the bottom-up effect more prominent [61]. This difference implies that the grass carp–crucian carp polyculture system relies more on internal nutrient recycling and redistribution, with lower dependence on external feed and fertilizers, which aligns well with the principles of ecologically healthy aquaculture.

The eDNA metabarcoding technique employed in this study identified a total of 288 plankton species, far exceeding the range of 50–120 species typically reported by conventional morphological surveys [22,30]. This revelation of “hidden diversity” benefits from the high sensitivity of high-throughput sequencing and its inherent independence from organismal developmental stages and morphological plasticity—species identification can be achieved from sloughed cells, mucus, or DNA in excretions even in the absence of intact individuals in the sample [31,33]. From an ecological perspective, these seemingly rare taxa with extremely low abundances may serve as functional hubs in certain key ecological processes, such as symbiotic associations between specific microbial groups and algae, or the buffering role of rare predators against dominant species [62]. However, the technical limitations of the approach should not be overlooked: the universal primers targeting the 18S V9 region exhibit relatively low amplification efficiency for groups such as diatoms, which may lead to systematic underestimation of their relative abundances [38]. Additionally, The 18S V9 primers used in this study may have led to underestimation of certain taxonomic groups, particularly diatoms, due to primer mismatches and differential amplification efficiency [63,64,65]. This bias may have influenced the observed seasonal patterns of diatom abundance, but since our core conclusions focus on overall community dynamics and comparative analyses across seasons and ponds (which used consistent protocols), the main findings remain robust; the incompleteness of reference databases—many microeukaryotic barcode sequences are still missing—rendered approximately 8.3% of the ASVs unassignable to the species level; and the degradation rate of eDNA is influenced by temperature, pH, UV radiation, and microbial activity [39]. Our observed winter decline in species richness (from 196–227 species in summer/autumn to 139 in winter) is consistent with the magnitude of seasonal declines reported in traditional morphological surveys of temperate aquatic ecosystems, suggesting that the signal primarily reflects ecological change rather than methodological artifacts.But, we need acknowledge a trade-off inherent to eDNA-based winter assessments: while low temperatures slow DNA degradation, they also suppress metabolic activity, reducing eDNA shedding rates [39,66]. Importantly, because the same primers and protocols were applied uniformly across all seasons, seasonal comparisons remain internally valid despite temperature-dependent effects on absolute eDNA concentrations. This interpretation is further supported by the observation that not all taxa declined uniformly in winter—*Chromulinospumella* increased dramatically during this season, confirming that eDNA detection remains effective when target organisms are present. We therefore focus our interpretations on relative seasonal shifts and compositional patterns rather than absolute abundance estimates. Nevertheless, the coverage values for all samples in this study exceeded 99.99% and all rarefaction curves reached a plateau, indicating that sequencing depth was not a limiting factor. Future improvements should focus on employing multi-marker gene combinations and constructing local reference databases to further enhance detection coverage and annotation accuracy.

Furthermore, the remarkably high proportion of positive edges (87.3%) in the species co-occurrence network warrants in-depth discussion. In classical ecological theory, competitive exclusion tends to generate negative correlations among species; however, the overwhelming predominance of positive correlations in this study suggests that interactions within the community are primarily governed by mutualism, niche complementarity, or shared environmental responses. We interpret these patterns as indirect, integrated responses to the combined effects of fish grazing and bioturbation, rather than as direct fish-plankton interactions. Co-occurrence networks are widely used in plankton ecology to infer the integrated outcome of environmental filtering, trophic interactions, and biotic associations, even when external drivers are not explicitly included as nodes [1,14,15,23]. The high proportion of positive edges and the central hub role of *Cryptomonas* are consistent with a facilitation-based framework in which fish-mediated disturbance—through top-down grazing and bottom-up nutrient release—creates heterogeneous microhabitats that promote positive associations among plankton taxa.

Several ecological explanations may account for this pattern: (1) Many phytoplankton species exhibit “facilitative effects”—for instance, extracellular polysaccharides secreted by certain algae can provide a carbon source for other heterotrophic microbes, which in turn mineralize organic matter and release inorganic nutrients that can be utilized by the algae, forming a reciprocal cycle [67]. In our dataset, several abundant taxa are known EPS producers. For example, *Cryptomonas*—which had the highest degree centrality [27] in our co-occurrence network—releases complex EPS that are degraded by heterotrophic bacteria, serving as a carbon source, while bacteria remineralize nutrients that support algal growth. Similarly, *Gonyostomum* and cyanobacteria present in our samples are also known to produce EPS and form mucilaginous aggregates that facilitate bacterial colonization. These taxon-specific patterns are consistent with the hypothesis that EPS-mediated mutualism contributes to the high proportion of positive correlations observed in our network; (2) Positive correlations may also arise from shared environmental preferences, whereby multiple species simultaneously favor the suitable temperature and nutrient conditions of autumn without engaging in intense resource competition, as niche differentiation is sufficiently pronounced to avoid competitive exclusion [68]; (3) The grazing activities of grass carp and crucian carp may act as a source of intermediate disturbance that promotes species coexistence. This disturbance arises from two complementary pathways. First, grass carp—while primarily herbivorous—exert spatially patchy grazing pressure on macrophytes and, when plant food is scarce, passively filter plankton, creating heterogeneous gaps in both macrophyte beds and the phytoplankton community. Second, crucian carp, as benthic omnivores, selectively graze on zooplankton while simultaneously resuspending sediments and releasing nutrients through bioturbation [69], generating a mosaic of disturbed and undisturbed microhabitats. This combination of direct consumption and physical disturbance operates at an intensity sufficient to suppress competitive dominants (e.g., large zooplankton and fast-growing phytoplankton) without eliminating sensitive taxa, thereby allowing more species to coexist than would be possible under either very low or very high disturbance regimes [70,71]. Importantly, the seasonal attenuation of fish activity in winter reduces disturbance intensity, consistent with the lower diversity observed during that season.

And, the community assembly mechanism analysis revealed that stochastic processes (particularly drift and dispersal limitation) contributed slightly more to community assembly than deterministic processes (56.3% vs. 43.7%), a ratio that is rather uncommon in eutrophic or highly controlled aquaculture systems. It is generally recognized that high-nutrient and high-productivity environments tend to favor deterministic processes (environmental filtering) as the dominant driver of community assembly [72,73,74,75]. The relatively high contribution of stochastic processes in this study may be related to the following factors: the niche space in the grass carp–crucian carp polyculture system is continually restructured by fish activity (sediment disturbance and pulsed nutrient release), generating highly heterogeneous microhabitat patches that facilitate stochastic colonization and the random assembly of local communities through dispersal and establishment. Although we did not directly measure nutrient concentrations, three lines of indirect evidence support the inference of pulsed nutrient release associated with fish activity. First, previous studies have demonstrated that fish excretion and fecal decomposition release significant amounts of nitrogen and phosphorus within hours to days following feeding or bioturbation events, creating ephemeral nutrient pulses [14,58,59]. Second, the seasonal patterns observed in our phytoplankton data—sharp increases following periods of intense fish activity—are consistent with episodic nutrient pulses rather than gradual accumulation alone. Third, nutrient budget studies in similar polyculture systems have reported that benthic fish can increase sediment-water nutrient fluxes by two- to three-fold through bioturbation [59]. Therefore, we frame our discussion of pulsed nutrient release as a mechanistic inference rather than a demonstrated conclusion. Direct measurements of dissolved nitrogen and phosphorus at fine temporal resolution would be required to confirm this mechanism. And, the particularly prominent contribution of stochastic processes in summer (67.2%) likely reflects the highly dynamic and stochastic nature of plankton communities during periods of rapid proliferation [76,77,78].

Comparing the results of this study with those of previous studies on monoculture or polyculture systems involving grass carp or crucian carp, several common patterns and differences emerge. Yang et al. [22] examined a grass carp + silver carp + common carp polyculture system and found that the phytoplankton community was dominated by green algae and diatoms, with cyanobacteria appearing during summer but not forming blooms. The presence of silver carp—a filter-feeding species that directly grazes on phytoplankton—in the Yang et al. system represents a key difference from our grass carp + crucian carp system, which lacks filter-feeding carp and relies on benthic foraging and bioturbation by crucian carp as the primary fish-mediated regulatory pathway. This compositional difference fundamentally alters the relative contributions of top-down versus bottom-up effects between the two systems. Despite this difference, both studies observed that cyanobacteria did not form blooms, suggesting that grass carp—common to both systems—may exert a certain inhibitory effect on cyanobacteria regardless of the associated species. The higher Shannon index observed in our study (mean 7.19) compared to previous pond studies may reflect both the ecological effect of polyculture and the higher sensitivity of eDNA metabarcoding. Our experimental design included both monoculture ponds (A2 and A3, stocked only with crucian carp fry) and polyculture ponds (A6 and A9, stocked with both grass carp and crucian carp). In July, when all four ponds were sampled, the Shannon index of polyculture ponds (mean 7.79) was notably higher than that of monoculture ponds (mean 6.19), suggesting that polyculture itself enhances plankton diversity—a finding consistent with Xiao et al. [79], who reported higher diversity in polyculture than in monoculture ponds using the same eDNA approach. However, the absolute Shannon values in our study are also higher than those reported in traditional morphological surveys [22,28], which likely reflects the higher sensitivity of eDNA metabarcoding in detecting rare or morphologically indistinguishable taxa [35,36,37]. Since our study did not include parallel morphological identification, we cannot quantitatively partition the contributions of polyculture effects versus eDNA sensitivity. We therefore focus our interpretations on relative seasonal patterns rather than absolute diversity values, and suggest that future studies integrating both eDNA and morphological approaches would be valuable to disentangle these two contributions. Li [80] also reported the highest phytoplankton diversity in autumn and the lowest in winter in grass carp ponds, a seasonal pattern consistent with our findings, indicating that seasonal influences on plankton are a universal phenomenon across regions and farming systems.

Our findings carry several practical implications for the management of grass carp–crucian carp polyculture ponds. First, the high plankton diversity and the predominance of positive interspecific interactions observed in this system suggest that polyculture itself functions as an effective ecological regulation strategy, reducing the risk of algal blooms through natural top-down and bottom-up controls. Second, the “stocked and left to feed naturally” regime—which relies entirely on natural food resources—appears to sustain a stable and diverse plankton community without external feed inputs, offering a low-cost and environmentally friendly alternative to intensive feed-based aquaculture. Third, the elevated stochasticity in community assembly during summer implies that ponds may respond differently to management interventions during warm months; therefore, routine monitoring of water color and transparency remains a practical tool for early detection of bloom risks. Finally, the seasonal attenuation of fish activity in winter, coupled with reduced plankton diversity, suggests that stocking densities or harvest timing could be adjusted seasonally to maintain ecological balance. These implications underscore the potential of grass carp–crucian carp polyculture as a sustainable model for large-water aquaculture.

## 5. Conclusions

This study represents a systematic application of eDNA metabarcoding to assess plankton biodiversity in a grass carp–crucian carp polyculture system, achieving a level of taxonomic resolution far exceeding that of previous studies. And, by incorporating community assembly mechanism analysis (null-model approach) into aquaculture ecology research, we disentangled the relative contributions of stochastic and deterministic processes, offering a new perspective for understanding the maintenance mechanisms of biological communities in aquaculture systems. Three theoretical constructs emerge from our findings. First, the balance between stochastic and deterministic processes shifts predictably with seasons—stochasticity dominates in summer (67.2%) when fish activity and metabolic rates peak, while deterministic selection becomes more important in winter, providing a temporal dimension to community assembly theory in managed aquatic systems. Second, grass carp and crucian carp jointly regulate plankton communities through spatially segregated but functionally complementary pathways—grass carp exert top-down control via filter-feeding in the upper water column, while crucian carp drive bottom-up nutrient regeneration through benthic foraging and bioturbation in the lower water column, with their combined effects sustaining high diversity through coupled feedback loops. Third, the high proportion of positive co-occurrences (87.3%) among plankton taxa challenges the conventional assumption that competitive exclusion dominates species interactions, and instead supports a facilitation-based framework in which EPS-producing taxa such as *Cryptomonas* serve as network hubs. Our findings not only demonstrate the broad application potential of eDNA technology for fine-scale biodiversity surveys in aquaculture ecology, but also provide direct molecular-level evidence for elucidating the ecological mechanisms underlying grass carp–crucian carp polyculture, thereby enriching the theoretical foundation for ecologically healthy large-water aquaculture. Future research should integrate multi-marker gene strategies, metatranscriptomics for functional profiling, high-frequency environmental monitoring, and mesocosm control experiments to further disentangle the quantitative relationships among fish grazing intensity, nutrient dynamics, and plankton community feedbacks, thereby providing a more robust scientific basis for scientifically formulating polyculture schemes, optimizing pond management, and ensuring the sustainable development of large-water fisheries.

## Figures and Tables

**Table 1:** PCR amplification system and thermal cycling program.

Amplification System	Amplification Procedure
Template: 1 μL	Initial denaturation at 95 °C for 3 min
Forward Primer (10 μM): 1 μL	Denaturation at 95 °C for 30 s
Reverse Primer (10 μM): 1 μL	Annealing at 55 °C for 30 s
2 × KAPA HiFi Mix: 15 μL	Extension at 72 °C for 15 s
ddH_2_O: up to 30 μL	Final extension at 72 °C for 5 min
Total volume: 30 μL	(25 cycles of denaturation, annealing, and extension)

**Table 2:** Alpha diversity indices of each sample.

Sample ID	Chao1	Shannon	Simpson	ACE
S20230715A2	1621	6.43	0.91	1622
S20230715A3	1522	7.64	0.98	1523
S20230715A6	1703	7.93	0.99	1703
S20230715A9	1093	4.74	0.85	1093
S20230830A6	1487	7.15	0.95	1486
S20230830A9	1775	7.57	0.96	1777
S20230916A2	2246	7.72	0.97	2247
S20230916A9	1403	7.85	0.98	1403
S20231018A2	1389	6.05	0.92	1390
S20231018A6	1719	8.13	0.99	1719
S20231018A9	1822	8.47	0.99	1823
S20231114A2	1662	6.75	0.95	1663
S20231114A9	1298	7.07	0.98	1299
S20231212A2	1445	6.72	0.97	1447
S20231212A6	1364	7.62	0.99	1365

## Data Availability

The original contributions presented in this study are included in the article. Further inquiries can be directed to the corresponding author (zyshen@hunnu.edu.cn).

## Supplementary Materials

The following supporting information can be downloaded at https://www.mdpi.com/article/10.3390/biology15171468/s1: Table S1: Taxonomic annotation of plankton at various classification levels.
