# Supplementary material for: Synergistic Regulation of Plankton Communities by Polyculture of Grass Carp and Crucian Carp: Seasonal Dynamics and Interaction Networks Based on eDNA
Source: Biology (Basel). 2026 Aug 30;15(17):1468. doi: 10.3390/biology15171468 (PMC13564559; doi:10.3390/biology15171468)
Supplement: Supplementary file 1 [file biology-15-01468-s001.zip › biology-4501748-supplementary.pdf]

Table S1. Taxonomic annotation of plankton at various classification levels.

Trophic level	Phylum	Class	Order	Family	Genus	Species
**First trophic level (primary producers)**	Alveolata	Dinophyceae	Thoracosphaerales	Thoracosphaeraceae	*Stoeckeria*	
	Stramenopiles	Bacillariophyta	Mediophyceae	Polar-centric-Mediophyceae		
				Stephanodiscaceae	*Discostella*	*Discostella_sp.*
			Coscinodiscophyceae	Radial-centric-basal-Coscinodiscophyceae		
				Aulacoseiraceae	*Aulacoseira*	*Aulacoseira_nyassensis*
				Stephanodiscaceae	*Cyclotella*	*Cyclotella_scaldensis*
				Skeletonemataceae	*Skeletonema*	*Skeletonema_subsalsum*
				Thalassiosiraceae	*Thalassiosira*	*Thalassiosira_tenera*
				Melosiraceae	*Melosira*	*Melosira_varians*
			Cymbellales	Gomphonemataceae	*Gomphonema*	
			Naviculales	Naviculaceae	*Navicula*	*Navicula_radiosa*
				Pinnulariaceae	*Pinnularia*	
				Stauroneidaceae	*Stauroneis*	
			Fragilariales	Fragilariaceae	*Synedra*	
		Chrysomerophyceae		Apoikiaceae	*Apoikiospumella*	*Apoikiospumella_mondseeiensis*
		Raphidophyceae	Chattonellales	Vacuolariaceae	*Gonyostomum*	*Gonyostomum_semen*
		Synurophyceae	Synurales	Mallomonadaceae	*Mallomonas*	*Mallomonas_caudata*
						Mallomonas_matvienkoae
						Mallomonas_striata
						Mallomonas_annulata
					Synura	
			Ochromonadales		*Acrispumella*	*Acrispumella_msimbaziensis*
		Xanthophyceae	Tribonematales	Tribonemataceae	*Heterothrix*	*Heterothrix_debilis*
			Mischococcales	Pleurochloridaceae	*Monodus*	
					Goniochloris	*Goniochloris_sculpta*
				Ophiocytiaceae	*Ophiocytium*	*Ophiocytium_capitatum*
		Actinophryidae		Oikomonadaceae	*Oikomonas*	*Oikomonas_sp*
	Chrysophyta	Chrysophyceae	Hibberdiales	Hibberdiaceae	*Chromophyton*	*Chromophyton_vischeri*
				Stylococcaceae	*Lagynion*	
			Chromulinales	Chromulinaceae	*Chromulinospumella*	*Chromulinospumella_sphaerica*
					Pedospumella	*Pedospumella_encystans*
					Chromulina_Clade-E	*Chromulina_chionophila*
					Spumella	*Spumella_lacusvadosi*
				Dinobryaceae	*Poteriospumella*	*Poteriospumella_lacustris*
					Epipyxis	*Epipyxis_pulchra*
					Poterioochromonas	*Poterioochromonas_malhamensis*
				Chrysocapsaceae	*Chrysosaccus*	*Chrysosaccus_sp.*
		Dictyochophyceae				
	Chlorophyta	Chlorodendrophyceae	Chlorodendrales	Chlorodendraceae	*Tetraselmis*	
		Chlorophyceae	Chaetophorales	Aphanochaetaceae	*Aphanochaete*	
				Chaetophoraceae	*Pleurococcus*	*Pleurococcus_sp.*
					Trichosarcina	*Trichosarcina_sp.*
				Schizomeridaceae	*Schizomeris*	*Schizomeris_leibleinii*
				Uronemataceae	*Uronema_Chlorophyceae*	
			Chlamydomonadales	Chlamydomonadaceae	*Carteria*	*Carteria_radiosa*
					Chlamydomonas	*Chlamydomonas_fasciata*
						Chlamydomonas_applanata
						Chlamydomonas_pseudomacrostigma
					Polytoma	
					Lobochlamys	*Lobochlamys_segnis*
					Spermatozopsis	*Spermatozopsis_similis*
				Golenkiniaceae	*Golenkinia*	*Golenkinia_longispicula*
				Chlorococcaceae	*Chlorococcum*	*Chlorococcum_vacuolatum*
				Haematococcaceae	*Gungnir*	*Gungnir_neglectum*
						Gungnir_kasakii
				Dunaliellaceae	*Hafniomonas*	*Hafniomonas_montana*
						Hafniomonas_reticulata
				Volvocaceae	*Pandorina*	*Pandorina_morum*
				Tetracystaceae	*Tetracystis*	*Tetracystis_pampae*
						Tetracystis_vinatzeri
				Stephanosphaeraceae	*Chlorogonium*	*Chlorogonium_capillatum*
			Chlorellales			
			Oedogoniales	Oedogoniaceae	*Oedogonium*	*Oedogonium_cylindrosporum*
			Sphaeropleales	Selenastraceae	*Monoraphidium*	
				Sphaeropleaceae	*Ankyra*	*Ankyra_judayi*
				Neochloridaceae	*Chlorotetraedron*	*Chlorotetraedron_incus*

				Scenedesmaceae	Desmodesmus	Desmodesmus_pannonicus
				Hydrodictyaceae	Monactinus	Monactinus_sturmii
				Mychonastaceae	Mychonastes	
				Radiococcaceae	Radiococcus	Radiococcus_sp
				Coccomyxaceae	Nannochloris	Nannochloris_bacillaris
			Chlorosarcinales	Chlorosarcinaceae	Neochlorosarcina	Neochlorosarcina_sp
				Nautococcaceae	Nautococcus	Nautococcus_solutus
				Phacotaceae	Phacotus	
					Pteromonas	Pteromonas_protracta
		Mamiellophyceae	Monomastigales	Monomastigaceae	Monomastix	Monomastix_minuta
		Nephroselmidophyceae	Nephroselmidales	Nephroselmidaceae	Nephroselmis	Nephroselmis_olivacea
		Pedinophyceae	Pedinomonadales	Pedinomonadaceae	Pedinomonas	Pedinomonas_minor
		Trebouxiophyceae	Chlorellales	Chlorellaceae	Closteriopsis	Closteriopsis_acicularis
					Muriella	
				Oocystaceae	Oocystella	Oocystella_oogama
					Oocystidium	Oocystidium_sp
					Chloroidium	
			unclassified_Trebouxiophyceae	Elliptochloris	Botryococcus	Botryococcus_terribilis
					Choricystis	
			Watanabea-Clade			
		Ulvophyceae	Cladophorales			
			Ulotrichales	Ulotrichaceae	Ulothrix	Ulothrix_zonata
	Cryptophyta	Cryptophyceae	Cryptomonadales	Hemiselmidaceae	Hemiselmis:nucl	Hemiselmis_sp.:nucl
				Hemiselmidaceae:nucl		
				Cryptomonadaceae	Cryptomonas	Cryptomonas_borealis
						Cryptomonas_gyropyrenoidosa
						Cryptomonas_obovoidea
						Cryptomonas_lundii
						Cryptomonas_marssonii
		Cryptophyceae:nucl	Cryptomonadales:nucl			
		Non	Pyrenomonadales	Chroomonadaceae	Chroomonas	Chroomonas_mesostigmatica
				Geminigeraceae	Teleaulax	Teleaulax_amphioxeia
						Teleaulax_acuta
		Goniomonadea	Goniomonadales	Goniomonadaceae	Goniomonas	Goniomonas_truncata
	Dinoflagellata	Dinophyceae	Gymnodiniales	Gymnodiniaceae	Nusuttodinium	Nusuttodinium_acidotum
						Nusuttodinium_aeruginosum
				Tovelliaceae	Esoptrodinium	Esoptrodinium_sp
					Tovellia	Tovellia_aveirensis
			Gonyaulacales	Ceratiaceae	Ceratium	
			Peridiniales	Peridiniaceae	Peridinium	
		Planomonadida				
	Euglenozoa	Euglenida	Aphagea	Distigmidae	Distigma	
			Heteronematales	Peranemidae	Anisonema	Anisonema_sp
					Peranema	Peranema_trichophorum
			Petalomonadida	Scytomonadidae		
					Petalomonas	Petalomonas_planus
	Glaucophyta	Glaucocystophyceae	Glaucocystophytes	Cyanophoraceae	Cyanophora	Cyanophora_paradoxa
	Haptophyta	Prymnesiophyceae	Coccolithales			
			Prymnesiales	Chrysochromulinaceae		
				Prymnesiaceae	Chrysochromulina	Chrysochromulina_tobin
		Pavlovophyceae	Pavlovales	Pavlovaceae	Pavlova	
	Ochrophyta	Eustigmatophyceae	Eustigmatales	Eustigmataceae	Characiopsis	Characiopsis_saccata
					Eustigmatos	Eustigmatos_sp.
				Monodopsidaceae	Nannochloropsis）	
			Goniochloridales		Pseudostaurastrum	
					Trachydiscus	Trachydiscus_minutus
				Chrysophyceae_Clade-C		
				Chrysophyceae_Clade-E		
				Chrysophyceae_Clade-F		
				Chrysophyceae_Clade_B2		
				Chrysophyceae_Clade-B1		
		Dictyochophyceae	Pedinellales		Pedinella	Pedinella_sp
					Pseudopedinella	Pseudopedinella_elastica
		Bacillariophyceae	Surirellales	Surirellaceae	Surirella	
	Rhodophyta	Bangiophyceae				
		Florideophyceae				
		Porphyridiophyceae	Porphyridiales	Porphyridiaceae	Porphyridium	Porphyridium_aerugineum
		Rhodellophyceae	Cyanidiales			
	Sagenista	Bicoecea	Bicoecales			
	Streptophyta	Angiosperms	Saxifragales	Haloragaceae	Myriophyllum	
					Mesostigma	Mesostigma_viride
		Zygnemophyceae	Zygnematales	Zygnemataceae	Spirogyra	
	Charophytes	Mesostigmatophyceae				
		Zygnemophyceae				
	Phaeophyta	Phaeophyceae				
	Euglenophyta	Euglenophyceae	Euglenales	Euglenaceae	Cryptoglena	Cryptoglena_longisulca
						Cryptoglena_similis

						Cryptoglena_skujae
						Cryptoglena_soropigra
					Euglena	*Euglena_adhaerens*
						Euglena_archaeoplastidiata
						Euglena_chadefaudii
						Euglena_laciniata
						Euglena_sp.
						Euglena_splendens
						Euglena_tristella
						Euglena_undulata
						Euglena_viridis
					Euglenaria	*Euglenaria_anabaena*
						Euglenaria_clavata
						Euglenaria_caudata
					Monomorphina	*Monomorphina_piriformis*
						Monomorphina_piropsis
						Monomorphina_pyrum
						Monomorphina_aenigmatica
					Strombomonas	*Strombomonas_triquetra*
						Strombomonas_verrucosa
						Strombomonas_balvayi
					Trachelomonas	*Trachelomonas_armata*
						Trachelomonas_conradi
						Trachelomonas_echinata
						Trachelomonas_hispida
						Trachelomonas_oblonga
						Trachelomonas_pertyi
						Trachelomonas_planctonica
				Phacaceae	*Lepocinclis*	*Lepocinclis_acus*
						Lepocinclis_cyclidiopsis
						Lepocinclis_helicoideus
						Lepocinclis_horridus
						Lepocinclis_oxyuris
						Lepocinclis_playfairiana
						Lepocinclis_steinii
						Lepocinclis_tripteris
					Phacus	*Phacus_circumflexus*
						Phacus_horeiformis
						Phacus_limnophila
						Phacus_mariae
						Phacus_paraorbicularis
						Phacus_platyaulax
						Phacus_pleuronectes
						Phacus_pusillus
						Phacus_raciborskii
						Phacus_ranula
						Phacus_rotunda
						Phacus_salina
						Phacus_sp.
						Phacus_trimarginatus
						Phacus_viridioryza
					Colacium	*Colacium_sp.*
	Bigyra	Bikosea	Pseudodendromonadales			
		Opalozoa	Bicosoecida	Bicoecaceae		
				Siluaniidae	*Adriamonas*	*Adriamonas_peritocrescens*
				Bicosoecidae	*Bicosoeca*	*Bicosoeca_petiolata*
				Araphid-pennate		
				Diplophrydaceae		
					Scherfellia	*Scherfellia_sp*
			Rhynosporida			
**Second trophic level (primary consumers)**	Amoebozoa	Archamoebea				
		Discosea-Flabellinia				
		Discosea-Longamoebia				
		Mycetozoa-Myxogastrea				
		Tubulinea	Arcellinida	Arcellidae	*Arcella*	*Arcella_hemisphaerica*
					Pyxidicula	*Pyxidicula_operculata*
				Cryptodifflugiidae	*Cryptodifflugia*	
			Echinamoebida	Echinamoebidae	*Echinamoeba*	*Echinamoeba_sp*
				Vermamoebidae		
					Vermamoeba	*Vermamoeba_vermiformis*
			Leptomyxida	Leptomyxidae		
			Mastigamoebida	Mastigamoebidae	*Mastigamoeba*	*Mastigamoeba_punctachora*
						Mastigamoeba_sp
		Variosea			*Filamoeba*	*Filamoeba_nolandi*

						ATCC50593-Flamella-WIM80-lineage
						Flamella-lineage
		Discosea	Centramoebida	Acanthamoebidae	Protacanthamoeba	Protacanthamoeba_sp
			Dactylopodida	Paramoebidae		
				Vexilliferidae	Vexillifera	Vexillifera_multispinosa
					Pseudoparamoeba	Pseudoparamoeba_pagei
			Himatismenida	Cochliopodiidae	Cochliopodium	
			Flabellinia	Vannellidae	Vannella	Vannella_miroides
						Vannella_simplex
					Paravannella	Paravannella_minima
					Ripella	Ripella_sp.
				Vexilliferidae		
				Paramoebidae	Korotnevella	Korotnevella_sp
						Korotnevella_stella
		Elardia	Arcellinida	Difflugiidae）		
			Leptomyxida	Leptomyxidae	Leptomyxa	Leptomyxa_reticulata
			Euamoebida	Hartmannellidae	Hartmannella	Hartmannella_cantabrigiensis
					Ptolemeba	Ptolemeba_bulliensis
	Alveolata	Dinophyceae	Colpodellida	Alphamonaceae	Alphamonas	Aphamonas_edax
			Suessiales	Suessiaceae	Asulcocephalium	Asulcocephalium_miricentonis
			Colponemida		Colponema	Colponema_sp.
	Stramenopiles	Opalinata				
		Pirsonia_Clade				
		Actinophryidae			Actinosphaerium	Actinosphaerium_eichhornii
			Pirsoniales		Pirsonia	
	Miozoa	Coccidiomorphea				
		Colponemea	Colponemida	Colponemidae		
	Apicomplexa	Colpodellidea				
		Gregarinomorphea				
		Conoidasida	Eimeriida			
			Eugregarinorida	Actinocephalidae	Hoplorhynchus	Hoplorhynchus_acanthatholius
				Gregarinidae		
				Leidyanidae	Leidyana2	Leidyana2_sp
				Monocystidae	Monocystis	Monocystis_agilis
			Neogregarinorida	Lipotrophidae	Mattesia	Mattesia_sp
			Eucoccidiorida	Aggregatidae	Aggregata	Aggregata_eberthi
				Sarcocystidae		
		Marosporida				
	Choanozoa	Corallochytrea	Corallochytrida			
	Centroheliozoa	Centroplasthelida	Pterocystida	Pterocystidae	Chlamydaster	Chlamydaster_sterni
				Raphidiophryidae	Raphidiophrys	Raphidiophrys_ambigua
						Raphidiophrys_contractilis
	Cercozoa	Sarcomonadea	Glissomonadida	Allapsidae	Allapsa	Allapsa_sp.
	Choanoflagellida	Choanoflagellatea	Craspedida	Monosigidae_Group_A		
				Monosigidae_Group_O		
				Salpingoecidae	Monosiga	Monosiga_ovata
				Salpingoecidae_Group_C2	Salpingoeca_C2	Salpingoeca_napiformis
			Corallochytrida	Corallochytridae		
		MAST-12	MAST-12C			
		MAST-2	MAST-2C			
		Ichthyosporea	Ichthyosphonida	Non	Anurofeca	
			Dermocystida	Rhinosporidiidae	Rhinosporidium	
	Chrompodellids					
	Ciliophora	Colpodea	Non	Bursariomorphida		
			Colpodida			
			Cyrtolophosidida	Platyophryida		
				Cyrtolophosididae	Cyrtolophosis	Cyrtolophosis_sp
				Woodruffiidae	Kuklikophrya	Kuklikophrya_ougandae
					Woodruffides	Woodruffides_metabolicus
			Bryometopida	Kreyellidae	Microdiaphanosoma	Microdiaphanosoma_arcuatum
		Heterotrichea	Heterotrichida	Blepharismidae	Blepharisma	
				Stentoridae	Stentor	
		Nassophorea	Nassulida	Furgasoniidae	Furgasonia	Furgasonia_blochmanni
		Oligohymenophorea	Peniculia			
			Scuticociliatia_2	Loxocephalidae	Dexiotricha	Dexiotricha_cf._granulosa
			Peniculida	Frontoniidae_1	Frontonia_1	Frontonia_leucas
						Frontonia_paramagna
				Frontoniidae_2		
				Lembadionidae	Lembadion	Lembadion_bullinum
				Parameciidae	Paramecium	Paramecium_polycaryum
			Pleuronematida	Histiobalantiidae	Histiobalantium	
			Urocentrida	Urocentridae	Urocentrum	
		Phyllopharyngea	Cyrtophoria_4			
			Suctoria	Evaginogenida	Heliophrya	Heliophrya_erhardi
			Chlamydodontida	Chilodonellidae	Chilodonella	
					Pseudochilodonopsis	Pseudochilodonopsis_quadrivacuolata
		Plagiopylea				
		Prostomatea	Prorodontida	Colepidae	Coleps	Coleps_hirtus
					Levicoleps	Levicoleps_biwae

				Holophryidae	*Pelagothrix*	*Pelagothrix_alveolata*
				Placidae	*Placus*	*Placus_striatus*
				Prorodontidae	*Prorodon*	*Prorodon_teres*
		Prostomatea_1				
		Prostomatea_2				
		Prostomatea_3				
		Spirotrichea	Choreotrichida			
			Hypotrichia			
			Tintinnida	Tintinnidiidae	*Tintinnidium*	
				Codonellidae	*Tintinnopsis_03*	*Tintinnopsis_lacustris*
			Sporadotrichida	Halteriidae	*Halteria*	*Halteria_grandinella*
				Oxytrichidae	*Histriculus*	*Histriculus_histrio*
					Oxytricha	*Oxytricha_granulifera*
					Stylonychia	*Stylonychia_notophora*
			Urostylida	Holostichidae		
				Bakuellidae	*Metaurostylopsis*	
				Urostylidae	*Diaxonella*	
					Paruroleptus	*Paruroleptus_lepisma*
					Uroleptus	*Uroleptus_gallina*
			Stichotrichida	Spirofilidae	*Pseudouroleptus*	*Pseudouroleptus_caudatus*
					Strongylidium	
				Oxytrichidae	*Cyrtohymena*	*Cyrtohymena_citrina*
		Odontostomatea	Odontostomatida	Epalxellidae	*Epalxella*	*Epalxella_antiquorum*
	Conosa					
	Discoba		Jakobida	Andaluciidae	*Andalucia*	*Andalucia_godoyi*
				Histionidae	*Reclinomonas*	*Reclinomonas_americana*
				Histionina	*Moramonas*	*Moramonas_marocensis*
					Seculamonas	*Seculamonas_ecuadoriensis*
			Thecamoebida		*Stenamoeba*	*Stenamoeba_amazonica*
	Euglenozoa	Diplonemea	Diplonemida	Diplonemidae	*Diplonema*	*Diplonema_ambulator*
						Diplonema_sp
				Hemistasiidae	*Hemistasia*	*Hemistasia_phaeocysticola*
		Kinetoplastea	Prokinetoplastida			
			Prokinetoplastina	Ichthyobodonidae	*Ichthyobodonidae_PRO1*	*Ichthyobodonidae_PRO1_sp*
			Trypanosomatida	Trypanosomatidae	*Leishmania*	
		Peranemea	Anisonemida	Anisonemidae		
	Foraminifera	Endomyxa	Plasmodiophorida	Polymyxa-lineage		
			Vampyrellida	Leptophryidae	*Platyreta*	*Platyreta_germanica*
		Endomyxa-Phytomyxea				
		Filosa-Granofilosea				
		Filosa-Imbricatea				
		Filosa-Sarcomonadea				
		Filosa-Thecofilosea				
		Monothalamids	Monothalamids_Clade-Y			
		Monothalamea		Reticulomyxidae	*Haplomyxa*	*Haplomyxa_saranae*
		Cercozoa	Cercomonadida	Cercomonadidae	*Cercomonas*	*Cercomonas_celer*
					Metabolomonas	*Metabolomonas_insania*
					Paracercomonas	*Paracercomonas_anaerobica*
			Cryomonadida	Rhogostoma-lineage		
				Rhogostomidae	*Rhogostoma*	*Rhogostoma_minus*
			Euglyphida	Euglyphidae	*Euglypha*	*Euglypha_filifera*
				Paulinellidae	*Paulinella*	*Paulinella_chromatophora*
			Limnofilida			
			Pansomonadida			
			Granofilosea	Clathrulinidae	*Clathrulina*	*Clathrulina_elegans*
				Limnofilidae	*Limnofila*	*Limnofila_borokensis*
						Limnofila_oxoniensis
			Nucleohelea			
			Glissomonadida	Allapsidae		
				Sandonidae		
			Imbricatea	Spongomonadidae	*Spongomonas*	*Spongomonas_minima*
	Hilomonadea		Rigifilida	Micronucleariidae	*Micronuclearia*	*Micronuclearia_podoventralis*
	Heliozoa		Desmothoracida			
	Lobosa	Heterolobosea	Tetramitia_V	Vahlkampfiidae_V	*Vahlkampfia*	*Vahlkampfia_avara*
	Metazoa					
	Metamonada					
	Nuclearida	Nucleariidae	Nucleariida		*Nuclearia*	*Nuclearia_thermophila*
	Protozoa	Preaxostyla				
			Planomonadidae			
	Annelida	Aphanoneura	Aeolosomatida	Aeolosomatidae	*Aeolosoma*	*Aeolosoma_sp.*
	Arthropoda	Chelicerata	Pycnogonida	Callipallenidae	*Austropallene*	*Austropallene_cornigera*
		Crustacea	Branchiopoda	Daphniidae	*Daphnia*	
			Malacostraca	Palaemonidae	*Macrobrachium*	*Macrobrachium_superbum（*
			Maxillopoda			
			Ostracoda	Cyprididae	*Strandesia*	*Strandesia_uenoi*
		Hexapoda	Diptera	Chironomidae	*Ablabesmyia*	*Ablabesmyia_rhamphe*
		Insecta				
		Copepoda	Cyclopoida	Cyclopidae	*Ectocyclops*	*Cyclopidae_sp.*
						Ectocyclops_polyspinosus
					Eucyclops	

	Bryozoa	Phylactolaemata	Plumatellida	Plumatellidae	*Plumatella*	*Plumatella_casmiana*
	Cnidaria	Myxozoa				
		Hydrozoa	Anthoathecatae	Hydridae	*Hydra*	
	Brachiopoda	Craniata				
	Gastrotricha		Chaetonotida	Chaetonotidae	*Lepidodermella*	*Lepidodermella_squammata*
	Mollusca	Bivalvia	Palaeoheterodonta			
			Unionida	Unionidae	*Elliptio*	*Elliptio_complanata*
		Gastropoda	Caenogastropoda			
			Heterobranchia			
			Sorbeoconcha	Cyclopsidae	*Cyclops*	
			Opisthobranchia	Elysiidae	*Hydropsyche*	*Hydropsyche_sp.*
	Nematoda	Chromadorea	Monhysterida	Monhysteridae	*Monhystera*	*Monhystera_paludicola*
		Enoplea				
					Prodontorhabditis	*Prodontorhabditis_wirthi*
	Platyhelminthes	Monogenea				
		Trematoda	Monopisthocotylea	Gyrodactylidae	*Gyrodactylus*	
		Turbellaria	Rhabdocoela			
		Digenea				
		Macrostomorpha	Macrostomida	Macrostomidae	*Macrostomum*	
			Catenulida	Stenostomidae	*Stenostomum*	*Stenostomum_leucops_aquariorum*
				Dalyelliidae	*Gieysztoria*	
				Typhloplanidae	*Olisthanella*	*Olisthanella_truncula*
	Rotifera					
	Myxosporidia	Myxosporea	Bivalvulida	Myxobolidae	*Myxobolus*	*Myxobolus_ampullicapsulatus*
						Myxobolus_nielii
				Myxidiidae	*Zschokkella*	
	Syndermata		Bdelloidea	Adinetidae	*Adineta*	*Adineta_vaga*
				Philodinidae	*Philodina*	*Philodina_megalotrocha*
		Monogononta	Ploimida	Gastropodidae	*Ascomorpha*	*Ascomorpha_ovalis*
				Brachionidae	*Notholca*	*Notholca_acuminata*
		Strombidiida				
		Vannellida				
			Acanthamoebidae			
			Agitatidae			
				Filamoebidae	*Flamella*	
				Moramonadidae		
				Paracercomonadidae		
				Paramastigidae		
			Ancyromonadida	Planomonadidae		*Planomonadidae_Group-2*
				Pseudoperkinsidae		
				Raphid-pennate		
				Sey055-lineage		
				TIN_03		
				Thecamoebidae		
					Monas	*Monas_sp*
			Ancyromonadida		*Nutomonas*	*Nutomonas_longa*
			Rigifilida		*Rigifila*	*Rigifila_ramosa*
					Syssomonas	*Syssomonas_multiformis*
				LOS7N/I-lineage		
				Rhynosporidae_Rhinosporidianae		
					Chlorochromonas	*Chlorochromonas_sp.*
					Group-Te	*Group_Te_sp.*
**Third trophic level (secondary consumers)**	Chordata	Mammalia				
		Osteichthyes	Teleostei			
		Gnathostomata	Primates	Hominidae	*Homo*	*Homo_sapiens*
		Actinopterygii	Cypriniformes	Cyprinidae	*Tinca*	*Tinca_tinca*
		Reptilia			*Tridentulus*	*Tridentulus_sp.*
**Fourth trophic level (saprophytic or parasitic decomposers)**	Amoebozoa	Eumycetozoa	Stemonitales-Physarales			
	Stramenopiles	Labyrinthulomycetes	Amphifilida	Amphifilaceae	*Fibrophrys*	*Fibrophrys_sp*
			Amphitremida		*Diplophrys*	*Diplophrys_sp*
		Oomycota	Peronosporales	Peronosporaceae	*Hyaloperonospora*	*Hyaloperonospora_parasitica*
					Phytophthora	
			Saprolegniales	Saprolegniaceae	*Achlya*	*Achlya_bisexualis*
					Saprolegnia	*Saprolegnia_sp.*
				Leptolegniaceae	*Aphanomyces*	*Aphanomyces_invadans*
			Lagenidiales	Lagenidiaceae	*Lagenidium*	*Lagenidium_giganteum*
			Pythiales	Pythiaceae	*Pythium*	
	Mesomycetozoa					

	Myxomycota	Myxomycetes	Physarales	Physaraceae		
			Fuscisporida	Physaridae	*Physarum*	*Physarum_cinereum*
	Pseudofungi					
	Eumycota					
	Ascomycota	Pezizomycotina	Sordariomycetes	Leotiomycetes	*Thelebolus*	*Thelebolus_stercoreus*
		Saccharomycotina	Saccharomycetales	Saccharomycodaceae	*Hanseniaspora*	
				Metschnikowiaceae	*Kodamaea*	
				Phaffomycetaceae	*Wickerhamomyces*	*Wickerhamomyces_anomalus*
		Dothideomycetes	Pleosporales	Pleosporaceae	*Cochliobolus*	
				Phaeosphaeriaceae	*Phaeosphaeriopsis*	
		Eurotiomycetes	Eurotiales	Trichocomaceae	*Aspergillus*	*Aspergillus_flavipes*
				Aspergillaceae	*Penicillium*	
	Basidiomycota	Agaricomycotina	Agaricomycetes			
			Tremellomycetes	Cryptococcaceae	*Cryptococcus*	*Cryptococcus_flavescens*
				Tremellaceae	*Tremella*	*Tremella_ramalinae*
				Trichosporonaceae	*Trichosporon*	
				Mrakiaceae	*Udeniomyces*	
		Pucciniomycotina	Microbotryomycete s	Sporidiobolales	*Rhodosporidium*	
					Sporobolomyces	*Sporobolomyces_inositophilus*
			Pucciniomycetes	Phragmidiaceae	*Gymnoconia*	*Gymnoconia_nitens*
				Melampsoraceae	*Melampsora*	
				Raveneliaceae	*Nyssopsora*	*Nyssopsora_echinata*
				Pucciniaceae	*Puccinia*	
		Ustilaginomycotina	Exobasidiomycetes	Microstromatales	*Jaminaea*	*Jaminaea_sp.*
				Entylomataceae	*Tilletiopsis*	*Tilletiopsis_pallescens*
			Ustilaginomycetes	Anthracoideaceae	*Schizonella*	
			Malasseziales	Malasseziaceae	*Malassezia*	
		Cystobasidiomycet es				
	Blastocladiomyco ta	Blastocladiomycoti na				
		Blastocladiomycete s	Blastocladiales	Catenariaceae	*Catenophlyctis*	
	Chytridiomycota	Chytridiomycotina				
		Chytridiomycetes				
		Rhizophydiomycet es	Rhizophydiales	Gorgonomycetaceae	*Gorgonomyces*	*Gorgonomyces_haynaldii*
				Rhizophydiaceae	*Rhizophydium*	*Rhizophydium_patellarium*
		Mesochytriomycete s	Gromochytriales	Gromochytriaceae	*Gromochytrium*	*Gromochytrium_mamkaevae*
	Monoblepharomy cota	Monoblepharidomy cetes	Monoblepharidales	Gonapodyaceae	*Monoblepharella*	
	Entomophthorom ycota	Entomophthoromy cotina				
		Entomophthoromy cetes	Entomophthorales	Ancylistaceae	*Conidiobolus*	*Conidiobolus_brefeldianus*
				Entomophthoraceae	*Pandora*	
	Mucoromycota	Mucoromycotina	Mucorales	Cunninghamellaceae	*Absidia*	*Absidia_sp*
				Mucoraceae	*Mucor*	
				Rhizopodaceae	*Rhizopus*	*Rhizopus_stolonifer_var._stol onifer*
